# Mitochondrial Respiration Inhibition Suppresses Papillary Thyroid Carcinoma *Via* PI3K/Akt/FoxO1/Cyclin D1 Pathway

**DOI:** 10.3389/fonc.2022.900444

**Published:** 2022-07-05

**Authors:** Bojie Chen, Shuwen Lei, Xinlu Yin, Mengjia Fei, Yixin Hu, Yuan Shi, Yanan Xu, Lei Fu

**Affiliations:** ^1^ Department of Head and Neck Surgery, Renji Hospital, School of Medicine, Shanghai Jiao Tong University (SJTU), Shanghai, China; ^2^ Shanghai Key Laboratory for Molecular Engineering of Chiral Drugs, School of Pharmacy, Shanghai Jiao Tong University, Shanghai, China; ^3^ SJTU-Agilent Technologies Joint Laboratory for Pharmaceutical Analysis, School of Pharmacy, Shanghai Jiao Tong University (SJTU), Shanghai, China; ^4^ Academy of Pharmacy, Xi’an Jiaotong-Liverpool University, Suzhou, China

**Keywords:** papillary thyroid carcinoma (PTC), mitochondria inhibition, respiratory chain, PI3K, Cyclin D1

## Abstract

**Background:**

Papillary thyroid carcinoma (PTC) is the most common thyroid malignancy, but little is known regarding PTC metabolic phenotypes and the effects of mitochondrial activity on PTC progression. The great potential of mitochondria-targeting therapy in cancer treatment promoted us to use tool compounds from a family of Mito-Fu derivatives to investigate how the regulation of mitochondrial respiration affected tumor progression characteristics and molecular changes in PTC.

**Methods:**

Mito-Fu L20, a representative of 12 synthetic derivatives, was chosen for mitochondrial inhibition experiments. Sample sections from PTC patients were collected and processed to explore potential molecular alterations in tumor lymph node metastasis (LNM). *In vitro* analyses were performed using human PTC cell lines (K1 and TPC-1), with the human normal thyroid follicular cell line (Nthy) as a control. K1 cells were injected into nude mice to generate an animal model. The mice were injected with normal saline or Mito-Fu L20 at 20 or 50 mg/kg every other day; their body weights and tumor volumes were also measured over time. To elucidate the resulting metabolic phenotype, we measured oxygen consumption rate (OCR) and extracellular acidification rate (ECAR), cellular adenosine triphosphate (ATP) levels and reactive oxygen species (ROS) production, and mitochondrial membrane potential. Wound healing and Transwell assays, cell cycle assays, real-time fluorescence quantitative PCR, Western blotting, and immunohistochemical staining were performed to explore glycolysis-dominant metabolism in PTC.

**Results:**

Cyclin D1 and mitochondrial complex IV were detected in tumor samples from PTC patients with LNM. Mito-Fu L20 showed dose-independent and reversible modulation of mitochondrial respiration in PTC. In addition to mitochondrial dysfunction and early apoptosis, G1/S phase arrest. Notably, reversible mitochondrial inhibition yielded durable suppression of tumor proliferation, migration, and invasion *via* the PI3K/Akt/FoxO1/Cyclin D1 pathway. *In vivo* experiments demonstrated that Mito-Fu L20 has a good safety profile and specific restorative effect on mitochondrial activity in the liver. In addition, Mito-Fu L20 showed antitumor effects, alleviated tumor angiogenesis, and improved thyroid function.

**Conclusion:**

Reversible inhibition of ATP production and durable suppression of PTC growth indicates that the downregulation of mitochondrial function has a negative impact on tumor progression and LNM *via* the PI3K/Akt/FoxO1/Cyclin D1 pathway. The results provide new insights into the antitumor potential and clinical translation of mitochondrial inhibitors.

## Introduction

Thyroid cancer is the most common endocrine malignancy worldwide; the most prevalent subtype is papillary thyroid carcinoma (PTC), the incidence of which has been steadily increasing for several years (1). PTC has a high rate of lymph node metastasis (LNM), the mechanism of which remains unclear; it is presumably associated with multiple genetic and epigenetic alterations, including BRAF^V600E^, TERT promoter, and phosphoinositol-3 kinase/AKT serine/threonine kinase (PI3K/Akt) pathway (2–5). In addition, only a few research groups have studied the metabolic phenotype of PTC to elucidate the associated molecular pathways and implications for both tumor proliferation and metastasis.

Mitochondria are the main site of energy production in all eukaryotic cells; they generate most cellular energy in the form of adenosine triphosphate (ATP) *via* oxidative phosphorylation (OXPHOS) (6). Respiratory chain complexes transfer electrons to complex IV, where electrons are further transferred into molecular oxygen, generating a proton gradient; complex V completes the production of ATP (6). In addition, the metabolites of OXPHOS act as second messengers, and such metabolites include reactive oxygen species (ROS), which participate in the cell cycle and apoptosis (7, 8). The amount of mitochondrial DNA (mtDNA) varies between normal and malignant tissues; mutation in mtDNA has been shown to trigger oncogenesis in approximately 30 cancers with different characteristics (6, 9). Functional mitochondrial energy production drives the progression of cancer cells, particularly in solid tumors. Furthermore, because of their poor blood supply and the resulting malnutrition, proliferating cells rely on mitochondria for energy; thus, mitochondrial function is a potential therapeutic target for antitumor drugs (6, 9, 10).

Triphenylphosphonium (TPP^+^) cation modifications have been shown to facilitate the targeting of mitochondria by various artificial compounds for the purpose of more potent inhibition of mitochondrial function; complex IV is especially susceptible to these effects (6). The Mito-Fu family (11), a series of TPP^+^-containing derivatives, has been reported to show antiaging and antiobesity effects, as well as a high cytotoxicity toward several tumor cell lines (4, 11, 12). For example, in HeLa cells, the Mito-Fu family has been shown to stimulate cellular ROS production and disrupt mitochondrial membrane potential, thereby promoting apoptosis and necrosis; thus, it functions as a mitochondrial inhibitor and a therapeutic antitumor agent (12). However, there is a need to characterize the specific genes or molecular pathways involved in manipulating mitochondrial function in tumors.

The present study was performed to investigate the mechanisms by which mitochondrial respiration regulates the progression of PTC; it also aimed to provide new insights into the clinical implementation of mitochondria-targeting agents. In this study, we explored the relationship between mitochondrial respiratory chain complex IV expression and cervical LNM in PTC patients, characterized the metabolic phenotype of PTC cell lines, validated the antiproliferative effects of novel TPP^+^-thiazole-derived compounds on mitochondria biogenesis as potential therapeutic interventions for PRC, and explored the corresponding alterations in relevant genes and molecular pathways both *in vitro* and *in vivo*.

## Materials and Methods

### Chemistry

#### General Procedures

All solvents and reagents were obtained from commercial suppliers and used without further purification. ^1^H Nuclear magnetic resonance (NMR) and ^13^C NMR spectroscopy were performed on Agilent 400- and 100-MHz NMR spectrometers (Agilent Technologies, Atlanta, GA, USA), respectively. Chemical shifts are expressed in parts per million (ppm). Coupling constants are shown in hertz (Hz). The splitting modes that described apparent multiplicities were designated as singlet (s), doublet (d), triplet (t), quartet (q), multiplet (m), and broad (br). High-resolution mass spectrometry (HRMS) and compound purity data were acquired on an Agilent 6200 TOF LC/MS system with UV detectors (220 and 254 nm). All reactions were monitored by thin-layer chromatography (silica gel, aluminum sheets 60 F254), which was performed by Qingdao Haiyang Chemical Co., Ltd. (Qingdao, Shandong, China). All crude products were purified by column chromatography using silica gel (200–300 mesh) that had been purchased from Qingdao Haiyang Chemical Co., Ltd.

**Scheme 1:**
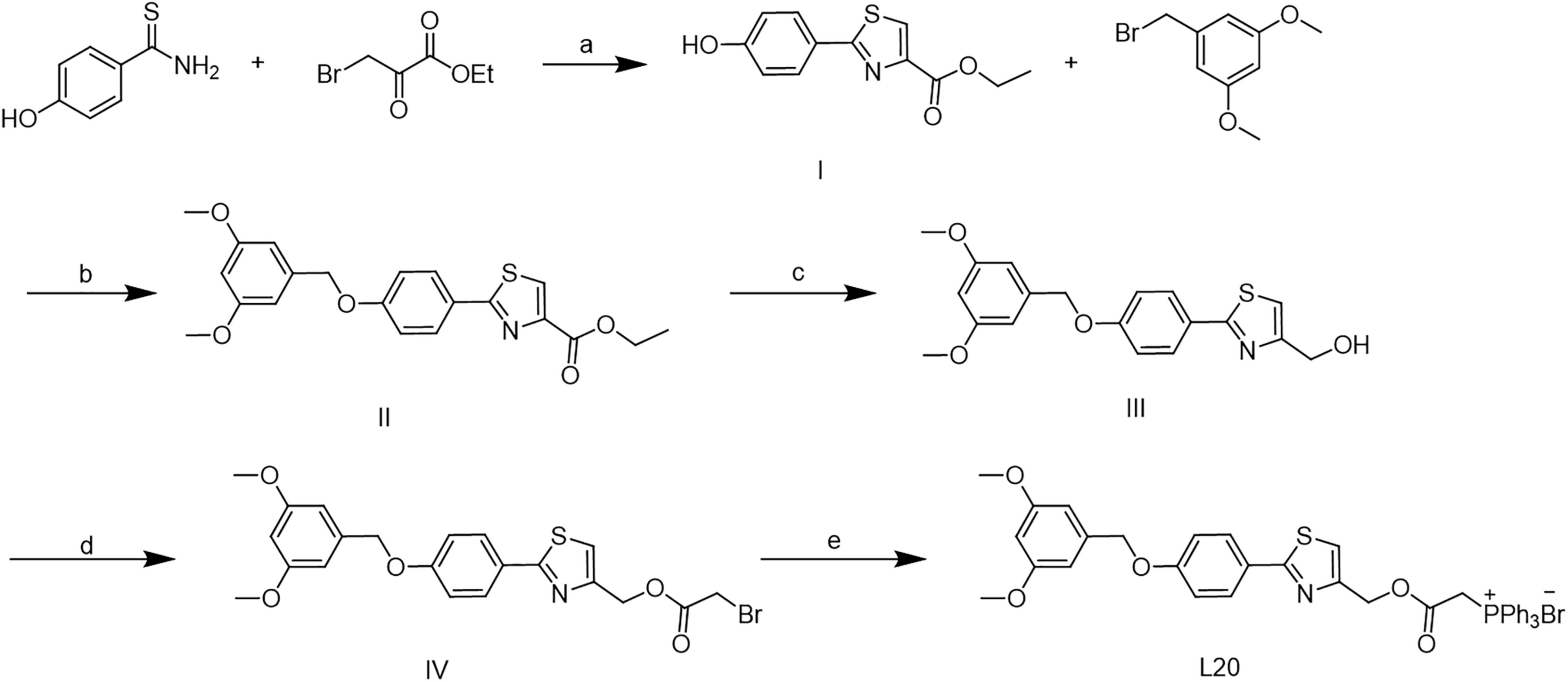
Synthesis of Mito-Fu L20. Reagent and conitions: (a) EtOH, 80 oC, overnight; (b) K2CO3, DMF, 80 oC, overnight; (c)LiAlH4, THF, r.t., 2 h; (d) 2- bromoacetyl bromide, DCM, r.t., overnight; (e) PPh3, toluene, r.t., overngiht.

The synthetic route of Mito-Fu L20 in shown in **Scheme 1**. Commercially available hydroxythiobenzamide (10 mmol) and ethyl 3-bromo-2-oxopropanoate (15 mmol) were dissolved in 30 ml of anhydrous ethanol; the reaction mixture was stirred continuously and refluxed overnight at 80°C to yield intermediate I. Intermediate I (5 mmol) was dissolved in 15 ml of N,N-dimethylformamide (DMF) with K_2_CO_3_ (7.5 mmol) and 3,5-dimethoxybenzyl bromide (7.5 mmol) at room temperature; the reaction mixture was stirred continuously and refluxed at 80°C overnight to yield intermediate II. Intermediate II was then dissolved in 20 ml of tetrahydrofuran; 12 mmol of LiAlH_4_ was added in a dropwise manner to the solution in an ice-water bath. The reaction mixture was stirred continuously at room temperature for 2 hours. After purification, intermediate III (6 mmol) was dissolved in 20 ml of DCM and 6 mmol of 2-bromoacetyl bromide was added to the solution in a dropwise manner in an ice-water bath; the reaction was continued with stirring overnight. A solution of 2 mmol of intermediate IV and 4 mmol of commercially available PPh_3_ was dissolved in 8 ml of toluene to yield Mito-Fu L20.

#### (2-((2-(4-((3,5-Dimethoxybenzyl)oxy)phenyl)thiazol-4-yl) Methoxy)-2- Oxoethyl) TPP^+^ Bromide (Mito-Fu L20)

White solid yields 63%. ^1^H NMR (400 MHz, DMSO-*d_6_*) δ 7.86–7.51 (m, 18H), 7.15 (d, *J* = 8.5 Hz, 2H), 6.65 (d, *J* = 2.3 Hz, 2H), 6.47 (t, *J* = 2.3 Hz, 1H), 5.55 (d, *J* = 14.4 Hz, 2H), 5.21 (s, 2H), 5.12 (d, *J* = 13.8 Hz, 2H), 3.74 (s, 6H). ^13^C NMR (100 MHz, DMSO-*d_6_*) δ 167.90, 164.82, 161.02, 160.44, 150.42, 139.48, 135.45, 134.19, 130.48, 128.16, 126.16, 119.62, 118.94, 118.06, 115.91, 105.95, 99.97, 69.78, 63.35, 55.67, 30.28.

HRMS(ESI) m/z C_39_H_35_NO_5_P_S_ calcd for [M]^+^ 508.1496 found: 508.1434

### Clinical Research

#### Patients and Clinical Information

A retrospective review was conducted involving patients who were hospitalized for surgical treatment in the Department of Head and Neck Surgery, Shanghai Jiao Tong University School of Medicine Affiliated Renji Hospital, between January 2020 and December 2020. The diagnosis of PTC was based on pathological features and included only patients with immunohistochemical (IHC) findings. Exclusion criteria were the treatment for other thyroid disorders such as hyperthyroidism or hypothyroidism, a history of thyroidectomy, a history of other malignant or rheumatoid diseases, and a treatment with immunosuppressive drugs. All records from each patient’s first admission were reviewed; we collected data regarding demographics, tumor characteristics, and IHC findings. The study protocol was approved by the Ethical Committee at Shanghai Jiao Tong University School of Medicine Affiliated Renji Hospital.

#### IHC Staining

Paraffin-embedded tissues were deparaffinized in xylene and rehydrated using a graded ethanol series. Endogenous peroxidase activity was blocked by immersion in 0.3% hydrogen peroxide in methanol; tissues were then incubated in 10% normal goat serum for 30 min. Subsequently, tissues were incubated for 30 min with antibodies to cytochrome c oxidase (DF7867, 1:100; Affinity BioReagents, Golden, CO, USA), Cyclin D1 (AF1183, 1:100; Beyotime Biotechnology, Shanghai, China), or FoxO1 (2880T, 1:100; Cell Signaling Technology, Beverly, MA, USA). Immunoreactivity was developed with diaminobenzidine (Dako, Glostrup, Denmark) for 5 min; tissues were then counterstained with hematoxylin. Tissues were viewed under a microscope (XSP-C204; Chongqing Optical Instrument Co., Ltd., Chongqing, China); three fields were randomly photographed at a magnification of ×200. The integrated optical density of each image was analyzed.

### Biology

#### Cell Culture

Human PTC cell lines (K1 and TPC-1) and the human normal thyroid follicular cell line (Nthy-ori 3-1) were obtained from the Cell Bank Affiliated to Shanghai Institute of Biochemistry and Cell Biology (Shanghai, China). Cells were maintained in Dulbecco’s modified Eagle’s medium (DMEM) supplemented with 10% fetal bovine serum (Thermo Fisher Scientific, Waltham, MA, USA) in a humidified incubator at 37°C with 5% CO_2_ in air.

#### Antiproliferation Assay

K1, TPC-1, and Nthy cells in logarithmic growth phase were inoculated into 96-well plates, and various concentrations of drugs were added to the wells; the wells were then incubated for an additional 24 h. Next, 20 μl of 3-(4,5-dimethylthiazol-2-yl)-2,5-diphenyltetrazolium bromide (Sigma-Aldrich Co., St. Louis, MO, USA) was added to each well and the cells were incubated for an additional 4 h. Then, 100 μl of dimethyl sulfoxide (DMSO) was added into all wells and the plates were shaken for 10 min. Finally, OD_570_ was measured using a microplate reader and results were expressed as values of IC_50_.

#### Mitochondrial Function Assessment by Seahorse XF

Cells were seeded at a density of 6000 cells per well on XF-96-well culture microplates. After various treatments, oxygen consumption rate (OCR) and extracellular acidification rate (ECAR) were measured (Seahorse XF96 Extracellular Flux Analyzer; Seahorse Bioscience, North Billerica, MA, USA) with a Mito Stress Kit (103015–100; Agilent Technologies) and Glycolysis Stress Kit (103020–100, Agilent Technologies) in accordance with the analyzer manufacturer’s protocol. For OCR measurement, 1.5 µM oligomycin, 0.5 μM carbonyl cyanide-4-(trifluoromethoxy)phenylhydrazone (FCCP), 1 μM rotenone, and 1 μM antimycin A (Rot/AA) were added sequentially, in accordance with the protocol provided by Agilent Technologies. The initial concentrations of certain agents (e.g., FCCP) were optimized, and starting seeding density was kept at 5,000 cells per well, respectively, according to the manufacturer’s protocol. For ECAR detection, 10.0 μM glucose, 1.0 μM oligomycin, and 50.0 µM 2-deoxyglucose (2-DG) were injected. Experimental treatments were performed with the wells of each plate as technical replicates. Basal OCR was calculated as [OCR_initial_ − OCR_Rot/AA_], ATP production was calculted as [OCR_initial_ − OCR_oligomycin_], basal ECAR was the result as [ECAR_Oligomycin_ − ECAR_Glucose_], and glycolytic capacity and glycolytic reserve were defined as [ECAR_2-DG_ − ECAR_Glucose_] and [ECAR_2-DG_ − ECAR_Oligomycin_], respectively.

#### Measurement of Cellular ATP Levels

Treated cells were lysed and subjected to analysis of total intracellular ATP content using an ATP assay kit (Beyotime Biotechnology) in accordance with the manufacturer’s instructions.

#### Determination of ROS Levels

After incubation and treatment, cells were washed three times with DMEM and then incubated in DCFH-DA staining solution provided with the Reactive Oxygen Species Assay Kit (Beyotime Biotechnology) at 37°C for 30 min in the dark. Subsequently, cells were washed twice with phosphate-buffered saline (PBS); fluorescence intensity was measured by flow cytometry (FACScan; BD Biosciences, Franklin Lakes, NJ, USA) with excitation and emission wavelengths of 488 and 525 nm, respectively.

#### Measurement of Mitochondrial Membrane Potential

Cells were seeded in six-well plates and subjected to various treatments, after which they were collected and loaded with the cationic carbocyanine dye JC-1 (5,5′,6,6′-tetrachloro-1,1′,3,3′-tetraethyl-imidacarbocyanine; Beyotime Biotechnology) for 30 min at 37°C in the dark; finally, they were washed twice with PBS and mitochondrial membrane potential was evaluated.

#### Wound Healing and Transwell Cell Migration Assays

For wound-healing assays, cells were cultured in 12-well plates and incubated overnight. After incubation for 24 h, the medium containing specific drugs was replaced with serum-free medium and the wells were scratched using 200-μl pipette tips. At 0 and 24 h after wounding, images were obtained using an inverted microscope (Nikon, Tokyo, Japan); wound width was measured using ImageJ software (National Institutes of Health, Bethesda, MD, USA).

For Transwell cell migration assays, cells were seeded in the upper chambers of 8-μm Transwells (Millipore, Billerica, MA, USA) and coated with Matrigel. After 48 h of incubation with serum-free medium and drugs, cells on the upper surface of the filter were removed; cells attached to the lower chambers were observed under an optical inverted microscope (Nikon).

#### Cell Cycle Assay

Fixed cells were centrifuged at 1,000 rpm and washed twice with cold PBS. RNase A (20 μg/ml) and propidium iodide staining solution (50 μg/ml) were added to the cells; incubation was then performed for 30 min at 37°C in the dark. Cells were analyzed by flow cytometry (FACScan; BD Biosciences) with CellQuest 3.3 software. FlowJo 10 software was used to determine the percentages of cells at different stages of the cell cycle.

#### Real-Time Quantitative PCR

Total RNA was extracted from cells using TRIzol (Invitrogen, Carlsbad, CA, USA). Single-stranded cDNA templates were obtained by reverse transcription using the First Strand cDNA Synthesis Kit (K1622; Invitrogen), and real-time quantitative PCR (RT-qPCR) was performed using the QuantityNova SYBR Green qPCR kit (208052; Qiagen, Germantown, MD, USA). Reaction conditions were 95°C for 120 s, followed by 45 cycles at 95°C for 5 s and 60°C for 10 s. Expression levels were normalized relative to β-actin; all measurements were performed in triplicate. Gene expression levels were using the 2^-ΔΔCt^ method. The amplification primer sequences were shown as follows: MTCO-1, 5′- TCCCCTAATAATCGGTGCCC-3′ (forward), 5′-GTTAGGTCTACGGAGGCTCC-3′ (reverse); MTCO-2, 5′-TCATGAGCTGTCCCCACATT-3’ (forward), 5′-TTGGTTTAGACGTCCGGGAA-3’ (reverse); MTCO-3,5’-ACCCACCAATCACATGCCTA-3′ (forward), 5′-CATTAGGAGGGCTGAGAGGG-3′ (reverse); and β-actin, 5′-GATCATTGCTCCTCCTGAGC-3′ (forward) and 5′-ACTCCTGCTTGCTGATCCAC-3′ (reverse).

#### Western Blotting

Cells were lysed and loaded onto 10% precast polyacrylamide gels (Mini-PROTEAN TGX Precast Gels; Bio-Rad, Hercules, CA, USA); they were then blotted onto polyvinylidene difluoride (PVDF) membranes (Trans-Blot Turbo Mini PVDF Transfer Packs; Bio-Rad). After the membranes had been blocked blocking Tween 20 in PBS containing 5% non-fat dry milk, they were incubated with primary antibodies against p-PI3K (abs130868; Absin Bioscience, Inc., Shanghai, China), p-AKT (4060S; CST, Danvers, MA, USA), FOXO (2880T; CST), and Cyclin D1 (AF1183; Beyotime) at 4°C overnight. Membranes were incubated with a horseradish peroxidase–onjugated secondary anti-rabbit (WB0177) and IgG (WB0176), as appropriate. Relative expression was determined using Image-Pro Plus software (v. 6.0; Media Cybernetics, Rockville, MD, USA).

#### *In Vivo* Experiment

Twenty-four BALB/c nude mice (4 weeks old, 18–20 g) were purchased from the Shanghai Experimental Animal Center (Shanghai, China) and randomly divided into four groups of six mice each. Six mice served as the control group, and other mice received a subcutaneous injection of were injected with 1×10^7^ K1 cells respectively by the left dorsal flank. Tumor-bearing mice received intraperitoneal injection of normal saline (NS) or the novel compound Mito-Fu L20 at 20 or 50 mg/kg every other day; the lengths and widths of the tumor masses were measured. The experimental protocol was approved by Animal Ethics Committee of Shanghai Jiao Tong University. The tumor volume was calculated as volume = (length × width^2^)×π/6. All tumor tissues were weighed before fixation in 4% paraformaldehyde; they were then embedded in paraffin and prepared for subsequent IHC analysis. Liver tissues were excised for quantitative detection of mitochondria (Beyotime), and peripheral blood was collected for further enzyme-linked immunosorbent assays (ELISAs) in accordance with the manufacturer’s instructions [Thyroid Stimulating Hormone (TSH), JL20301, Thyroglobulin (Tg) JL11109, vascular endothelial growth factor (VEGF) JL11063; J&L Biological, Shanghai, China].

### Statistical Analysis

Statistical analyses were performed using SPSS software (v.16.0; SPSS Inc., Chicago, IL., USA). Continuous variables were expressed as means ± standard deviations (SDs), and categorical variables were expressed as frequencies and percentages. Means were compared between groups using Student’s *t*-test, the Mann–Whitney *U* test, and one-way analysis of variance (ANOVA) as appropriate, according to the number of groups and normality of the data distribution. For categorical comparisons, the chi-square test was used. In all analyses, a two-sided *P*-value < 0.05 was considered indicative of statistical significance.

## Results

### Clinical Characteristics of Patients With PTC

**Figure 1:**
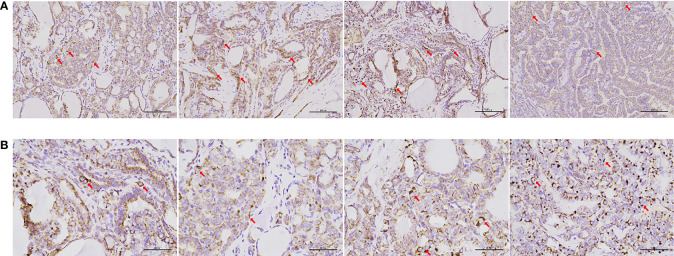
IHC of cytochrome C oxidase in samples from patients with PTC. Complex IV expression was detected in most surgical specimens of papillary thyroid carcinoma with central or lateral lymph node metastasis. **(A)** Immunohistochemistry, 20× (scale bar: 100 μm). **(B)** IHC, ×40 (scale bar: 50 μm). Cells and areas indicated by red arrows are highly pigmented, indicating high levels of cytochrome c oxidase expression.

A total of 318 patients (212 women and 106 men; mean age, 43.38 ± 13.82 years) who underwent surgical treatment for PTC at our center in 2020 were included in this study. The mean maximum tumor diameter was 15.55 ± 7.26 mm; 238 (75.2%) cases were unifocal and 256 (80.4%) cases were unilateral. All surgical specimens were collected from paraffin-embedded surgical tissues after patients had provided informed consent. In comparison to patients without LNM, patients with central or lateral LNM were more likely to exhibit positive staining for Cyclin D1 (**Table 1**); in these specimens, complex IV staining was strongly positive with average optical density of 0.26 ± 0.02 (**Figure 1**). Therefore, cell cycle dysregulation may promote LNM of PTC, which would involve mitochondrial biogenesis.

**Table 1:** Clinicopathological variables and IHC findings in PTC in relation to (central) lymph node metastasis.

	With central LNM (n = 164)		With no central LNM (n = 154)		*P* value	With LNM (n = 172)		With no LNM (n = 146)		*P*-value
	Missing	Mean ± SD, Positive (%)	Missing	Mean ± SD, Positive (%)		Missing	Means ± SD, Positive (%)	Missing	Means ± SD, Positive (%)	
**Age**	–	41.43 ± 13.52	–	45.45 ± 13.87	0.009	–	41.47 ± 13.54	–	45.64 ± 13.84	0.007
**Gender**					0.429					0.383
Male	–	58 (35.4%)	–	48 (31.2%)		–	61 (35.5%)	–	45 (30.8%)	
** F**emale	–	106 (64.6%)	–	106 (68.8%)		–	111 (64.5%)	–	101 (69.2%)	
**Tumor features**										
Unifocal	–	115 (70.1%)	–	123 (79.9%)	0.045	–	120 (69.8%)	–	118 (80.8%)	0.024
Unilateral	–	125 (76.2%)	–	131 (85.1%)	0.047	–	131 (76.2%)	–	125 (85.6%)	0.034
Diameter (mm)	–	16.95 ± 8.17	–	14.06 ± 5.81	<0.001	–	17.06 ± 8.24	–	13.77 ± 5.42	<0.001
**IHC**										
BRAF^V600E^	0	147 (89.6%)	2	142 (93.4%)	0.229	0	151 (87.8%)	2	134 (93.1%)	0.352
Cyclin D1	48	112 (96.6%)	33	90 (74.4%)	<0.001	49	117 (95.1%)	32	83 (72.8%)	<0.001
Galactin-3	54	102 (92.7%)	67	79 (90.8%)	0.624	60	106 (94.6%)	61	77 (90.5%)	0.564
TTF-1	10	147 (95.5%)	7	138 (93.9%)	0.542	10	154 (95.1%)	7	130 (93.5%)	0.406
P53	80	45 (53.6%)	67	53 (60.9%)	0.331	83	47 (52.8%)	64	49 (59.8%)	0.535

### *In Vitro* Biological Evaluation and Pathway Exploration

The antiproliferative capacities of the novel compounds against thyroid cell lines *in vitro* are shown in **Table 2**. Mitochondria-targeting inhibitors significantly reduced the rate of cell growth. The IC_50_ values of Mito-Fu L20 were low in K1 and TPC-1 cells but relatively high in Nthy cells, indicating ideal cytotoxicity and tumor specificity. Therefore, Mito-Fu L20 was regarded as an ideal compound for manipulation of tumor mitochondria; it was selected for subsequent experiments in this study.

**Table 2:** Antiproliferative effects of novel compounds Mito-Fu L1-L20 on normal thyroid and papillary thyroid carcinoma cell lines.

IC_50_ (μM) Calculation.			
Compound index	Nthy	TPC-1	K1
**L1–L8**	>50	>50	>50
**L9**	17.1 ± 1.8	14.0 ± 2.8	11.9 ± 1.8
**L10**	23.8 ± 1.8	24.9 ± 0.7	17.3 ± 2.1
**L11**	16.6 ± 1.0	16.8 ± 1.7	11.9 ± 1.3
**L12**	9.7 ± 0.6	13.7 ± 2.2	11.0 ± 0.7
**L13**	>50	>50	>50
**L14**	15.2 ± 1.9	16.7 ± 2.8	13.1 ± 1.7
**L15**	25.0 ± 1.7	21.1 ± 1.6	20.2 ± 1.0
**L16**	>50	>50	>50
**L17**	35.8 ± 1.8	33.1 ± 1.3	>50
**L18**	>50	>50	>50
**L19**	15.5 ± 0.6	17.1 ± 0.2	10.2 ± 0.2
**L20**	15.9 ± 1.7	11.6 ± 1.2	11.7 ± 0.6

Values were means of triplicates, and all experiments were performed for three times.

**Figure 2:**
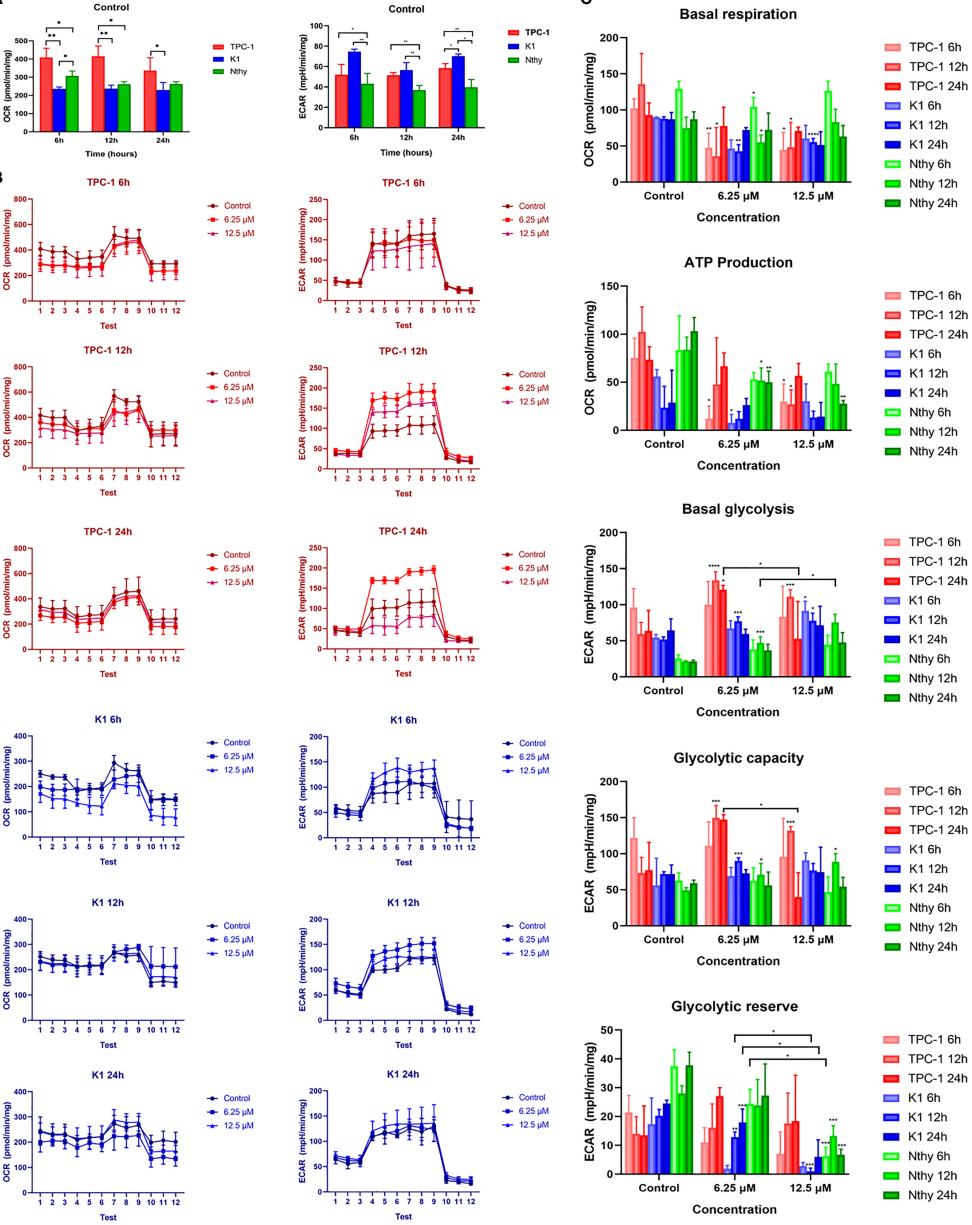
Metabolism phenotypes of normal thyroid follicles and papillary thyroid carcinoma cells with and without mitochondrial inhibition. The metabolic phenotypes of the PTC cell lines (K1 and TPC-1) differed in terms of baseline OCR (pmol/min/mg) and ECAR (mpH/min/mg) **(A)**. TPC-1 cells had a significantly higher OCR and significantly lower ECAR, compared with K1 and Nthy cells. Three sequential tests were performed after three injections of the corresponding drugs: oligomycin, FCCP, and Rot/AA for OCR determination; glucose, oligomycin, and 2-DG for ECAR determination. The dynamics of the metabolic phenotype are shown **(B)**. To further assess the effect of Mito-Fu L20 on mitochondrial function, basal respiration, ATP production, basal glycolysis, glycolytic capacity, and glycolytic reserve were calculated and compared **(C)**. Mito-Fu L20 significantly reduced ATP production in PTC cells (TPC:6 h, 6.25 μM and 12.5 μM, P < 0.05; 12 h, 12.5 μM, P < 0.05) (K1: 6.25 μM, P < 0.05) and consequently inhibited basal respiration (TPC: 6 h, 6.25 μM, P< 0.01; 12.5 μM, P < 0.05; 12 h, 6.25 μM and 12.5 μM, P < 0.05) (K1: 12 h, 6.25 μM, P < 0.01; 12.5 μM, P < 0.001). Mito-Fu L20 increased basal glycolysis levels in PTC cells but did not show a consistent effect on the glycolytic capacity of tumors. However, Mito-Fu L20 disrupted the glycolytic reserve capacity of PTC cells, which reflected a loss of the ability of those cells to meet their energy requirements, especially in K1 cells (12 h, 6.25 μM, P < 0.01; 12.5 μM, P < 0.005; 24 h, 6.25 μM, P < 0.005). *P < 0.05, **P < 0.01, ***P < 0.005, ****P < 0.001. Values are means of triplicate samples, and all experiments were performed at least three times.

The metabolic phenotypes and mitochondrial functions of thyroid cell lines before and after Mito-Fu L20 intervention are shown in **Figure 2**. PTC cell lines showed varied OCR (pmol/min/mg) and ECAR (mpH/min/mg) compared to each other and to normal thyroid follicular cells. Mitochondrial respiration was more dominant in TPC-1 cells, whereas K1 cells relied on glycolysis at a basal level (**Figure 2A**); these results suggested that the BRAF^V600E^ mutation may alter the metabolic pattern of PTC. Similar to many other tumor types, PTC cells displayed an altered metabolic phenotype in terms of glycolysis. After treatment with Mito-Fu L20 at different concentrations and for different periods, PTC cells showed a significant reduction in basal respiration and ATP production (**Figures 2B, C**). In addition, Mito-Fu L20 also showed modulation of basal glycolysis, glycolytic capacity, and glycolytic reserve in PTC cells. Moreover, these alterations associated with Mito-Fu L20 treatments were evident in the groups treated with lower drug concentrations or for shorter periods, suggesting that the inhibition of mitochondrial respiration was reversible.

**Figure 3:**
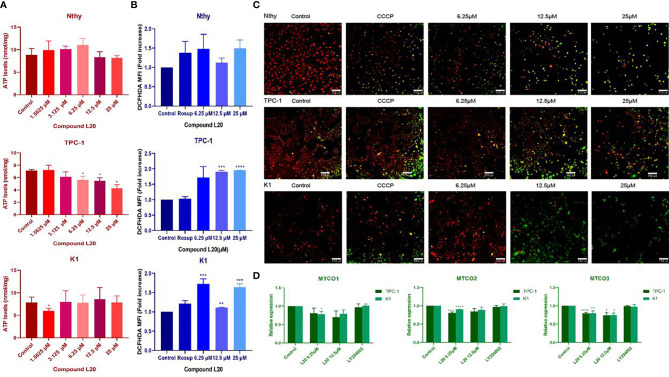
Effects of Mito-Fu L20 on mitochondrial biogenesis in normal thyroid cells and papillary thyroid carcinoma cells. Treatment with different concentrations of Mito-Fu L20 significantly altered **(A)** cellular ATP levels, **(B)** ROS production, **(C)** mitochondrial membrane potential, and **(D)** gene expression of complex IV subunits in papillary thyroid carcinomas (TPC-1 and K1 cells). **(A)** ATP levels were significantly decreased in treated TPC-1 cells, in a dose-dependent manner. **(B)** ROS production in PTC was elevated after treatment with Mito-Fu L20, except in the K1 group treated with 12.5 μM Mito-Fu L20. **(C)** Red signals indicate normal cells and green signals indicate apoptosis. Thus, in the overlapping images, the yellow and red signals indicate normal cells, whereas the green signals indicate apoptosis. The higher concentration of Mito-Fu L20 was chosen as the intervention, and more green signals were detected (scale bar: 150 μm). **(D)** Expression levels of complex IV subunits, including MTCO1, MTCO2, and MTCO3 were determined by RT-qPCR. These genes were significantly inhibited after Mito-Fu L20 intervention, but inhibitory effects for MTCO3 were found only with an increased drug concentration. *P < 0.05, **P < 0.01, ***P < 0.005, ****P < 0.001. Values are means of three replicates, and all experiments were performed at least three times.

Mito-Fu L20 treatment significantly affected mitochondrial biogenesis in the PTC cell lines (TPC-1 and K1), including marked decreases in ATP production (**Figure 3A**), and significant increases in ROS levels (**Figure 3B**). Cells were labeled to detect mitochondrial membrane potential. Under normal conditions, cells were labeled with a red signal, while cells in the early apoptosis were labeled with a green signal. PTC cells with impaired mitochondrial function appeared likely to undergo early apoptosis (**Figure 3C**).

Downregulation of the major subunits of complex IV (MTCO1, MTCO2, and MTCO3) was detected by RT-qPCR, which confirmed the targeting ability of Mito-Fu L20 (**Figure 3D**).

**Figure 4:**
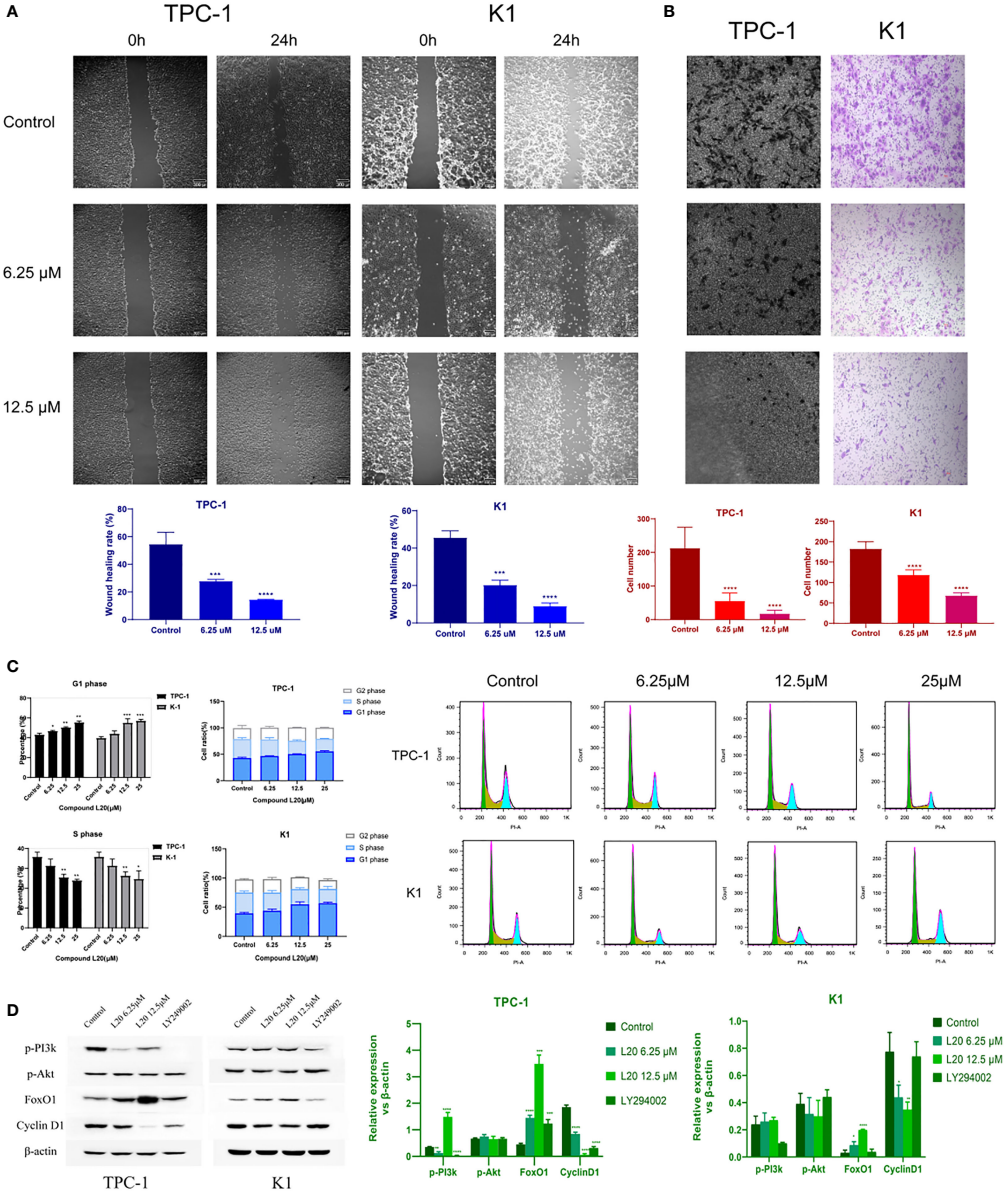
Mito-Fu L20 attenuated PTC migration and invasion, delayed the tumor cell cycle, and inhibited the PI3K/Akt pathway. **(A)** Wound healing assays were performed to examine cell migration capacity (scale bar: 300 μm). **(B)** Invasive capacities of PTC cells were measured by Transwell assays (scale bar: 300 μm). **(C)** Flow cytometry and **(D)** Western blotting analysis were used to detect changes in the expression patterns of proteins involved in the PI3K/Akt/FoxO/Cyclin D1 pathway and changes in the cell cycle in treated normal thyroid follicles and PTC cells. *P < 0.05, **P < 0.01, ***P < 0.001, ****P < 0.0001. Values are means of three replicates, and all experiments were performed at least three times.

After mitochondrial modification, the migration and invasion of PTC cells were inhibited, indicating a potential for tumor suppression (**Figures 4A, B**). Further exploration of the mechanisms underlying the tumor suppressive effects of mitochondrial L20 treatment indicated that the G1/S retardation in the intervention groups was dose-dependent (**Figure 4C**).

The genes and pathways involved in tumor metabolism, invasion, migration, and cell cycle regulation were presumed to be significantly affected by impaired mitochondrial biogenesis. Therefore, the expression levels of relevant genes were measured; the result showed that FoxO1 was significantly upregulated, while Cyclin D1 was significantly downregulated after mitochondrial inhibition in tumor cells. Significant inhibition of p-PI3K was also observed inTPC-1 cells treated with Mito-Fu L20 at 6.25 µM, but a surprising rebound was observed at a concentration of 12.5 µM (**Figure 4D**).

### *In Vivo* Validation of Biosafety, Antitumor Effects, and Further Exploration

**Figure 5:**
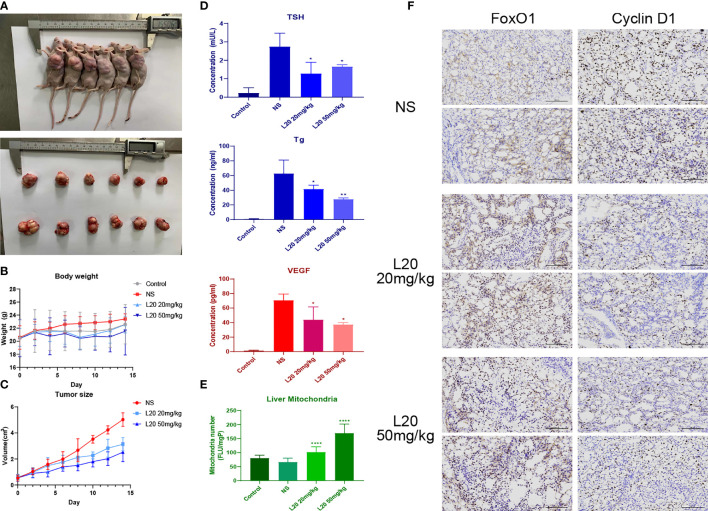
Tumor-suppressive effects of Mito-Fu L20 and its effects on thyroid-related indicators. Treatment with Mito-Fu L20 in a subcutaneous tumor animal model. Mito-Fu L20 was injected intraperitoneally, followed by measurements of body weight and tumor volume every other day **(A)**. Tumors removed from nude mice on day 14. **(B)** Body weight and **(C)** tumor size were measured every other day; tumor volumes were calculated using the ellipsoid formula: V = (length × width^2^) × π/6. **(D)** Levels of TSH, Tg, and VEGF were measured in peripheral blood samples. **(E)** Quantification of the number of mitochondria in liver tissue and comparison between the NS and drug treatment groups. **(F)** Tumor specimens were subjected to IHC staining (scale bar: 100 μm). Data are shown as means ± SDs (n = 6 for each group). *P < 0.05, **P < 0.01, ***P < 0.005, ****P < 0.001. NS, Normal saline.

The body weight and tumor size of BALB/c nude mice were measured every other day, along with the intraperitoneal injection of NS or Mito-Fu L20 at concentrations of 20 and 50 mg/kg (**Figure 5A**). Beginning at the fifth intervention, significant decreases in tumor volume were found in the Mito-Fu L20 group (**Figure 5B**).

TSH is an important reference factor for assessing thyroid function, whereas Tg is presumed to predict postoperative recurrence of PTC. ELISAs showed that the disruption of mitochondrial function in experimental groups resulted in decreased levels of TSH and Tg compared to the NS group (**Figure 5C**). Similar inhibitory effects were also observed with regard to VEGF levels (**Figure 5D**), suggesting that the inhibition of mitochondrial respiration reduced tumor angiogenesis.

The mitochondrial contents in liver slices from each group of mice were further quantified. The fluorescence (FLU) generated per well was recorded and the total FLU per milligram of protein (FLU/mgP) was determined using excitation and emission wavelengths of 520 and 590 nm, respectively. Surprisingly, the Mito-Fu L20 treatment groups showed greater preservation of liver mitochondria, compared to the NS group (**Figure 5E**).

In the Mito-Fu L20 interventions groups, FoxO1 expression was upregulated, whereas Cyclin D1 expression was significantly downregulated. The average optical density showed significant differences between the NS and treatment groups (**Figure 5F**).

In conclusion, Mito-Fu L20 inhibited the respiratory chain, specifically by targeting complex IV *in vivo*; it also altered and optimized mitochondrial functions in various organs and tissues. The altered mitochondrial biogenesis also led to altered thyroid function, further supporting the complex anticancer effects of this mitochondria-targeting agent.

## Discussion

The results of this study suggest that alteration of the metabolic phenotype of PTC by mitochondria-targeting agents, particularly in terms of ATP production efficiency, can influence tumor suppression; the effects include the inhibition of tumor proliferation, migration, and invasion, along with obstruction of the cell cycle and acceleration of apoptosis *via* the PI3K/Akt/FoxO1/Cyclin D1 pathway. In addition, mitochondria-targeting drug therapy alleviated tumor angiogenesis and improved thyroid function, suggesting multiple uses in treating PTC.

Although surgery is the first choice for PTC treatment, a subset of patients with persistent/recurrent/metastatic PTC (prm-PTC) or radioiodine refractory PTC (RAIR-PTC) requires systematic therapy. Thus, there is a need for further research regarding adjuvant drugs (10, 11). A few drugs (e.g., lenvatinib) have been approved for clinical use in RAIR-PTC that is locally advanced, metastatic, symptomatic, life-threatening, unsuitable for local treatment, or continues to progress 12 months after radical treatment (11, 12). Moreover, multiple drug candidates are currently in clinical trials, including cabozantinib (13); the pathways and receptors targeted by these drugs mainly include the RAF/MEK/ERK pathway, VEGFR, and PDGFR.

A literature review showed that the antitumor potential of mitochondrial interventions in PTC has rarely been investigated, presumably because of limited knowledge concerning the altered metabolic phenotype of PTC. Under physiological conditions, cells preferentially utilize OXPHOS when sufficient oxygen is available; they switch to anaerobic glycolysis under oxygen-limited conditions (13). However, the Warburg effect (i.e., aerobic glycolysis) implies that the dominant metabolic phenotype of cells is glycolysis even under conditions of oxygen sufficiency in many cancers; this effect is also present in some proliferative diseases with malignant features (13, 14). In the present study, the Warburg effect was also observed in PTC. Moreover, the balance between mitochondrial respiration and anaerobic glycolysis varied between PTC cell lines, suggesting that the BRAF^V600E^ mutation plays a key role in tumor metabolism (3, 15). This study provides new insights into the combined of drugs with different targets for widely studied molecules and genes as well as key links in metabolism.

Mitochondria-related therapeutics have been extensively studied (16); TPP^+^, which has three phenyl groups and a single positive charge delocalized over these groups, is widely recognized as one of the most effective mitochondria-targeting cations (6, 17). Generally, its stable resonance structure contributes to its high affinity for the lipophilic phospholipid bilayers of organelles, such as mitochondria (17). TPP^+^ can target mitochondria in cancer cells because their membrane potential (−220 mV) is significantly lower than that of normal cells (18). The novel compound, Mito-Fu L20 [2-(2-(4-methylthiazol-5-yl) ethoxy-2-oxoethyl triazole], is reportedly effective in altering mitochondrial function, delaying aging, correcting metabolic imbalances, and reversing the progression of metabolic syndrome both *in vivo* and *in vitro* (4). Furthermore, the effects of long-term Mito-Fu intake on the levels of TNF-α, pAMPK, FoxO1, and other genes or proteins prompted us to investigate its effects on typical tumor-related pathways (4, 18). In addition, the antitumor effects of novel TPP^+^-thiazole derivatives were validated in our previous study, which revealed modulation of mitochondrial complex IV (18). It was suggested by mRNA sequence analysis and gene enrichment results from the Gene Ontology and Kyoto Encyclopedia of Genes and Genomes databases that our novel compound significantly altered several key pathways in the endocrine system and significantly delayed the cell cycle in tumor cells, which prompted us to further explore its potential mechanisms of action.

The expression levels of components of the PI3K/Akt pathway and downstream molecules, such as FoxO1 and Cyclin D1, were significantly affected by treatment with mitochondria-targeting inhibitors. However, in contrast to the significant changes in FoxO1 and Cyclin D1 expression, Mito-Fu L20 had an apparently small effect on the phosphorylation of PI3K and Akt. This finding suggested the potential for another important pathway through which mitochondrial biogenesis can regulate FoxO1 and its related genes (19). Previous studies have explored the communication between the PI3K/Akt pathway and mitochondrial biogenesis (20); the PI3K/Akt pathway induces mitochondrial reprogramming in melanoma, prostate cancer, and many other tumors (21, 22). Akt2, a subunit of Akt, markedly affects the posttranscriptional modification of Cyclophilin D (CypD), which is an essential component of mitochondrial permeability transition pores; changes in this modification lead to altered CypD activity and mitochondria membrane permeability (23). Mitochondrial reprogramming is involved in stress-related cell death, promotes tumor cell survival, provides the energy for tumor migration and invasion, and enhances drug resistance in tumor cells (21, 24–26). The combination of PI3K therapy with drugs that inhibit mitochondrial biogenesis has been shown to significantly suppress mitochondrial fitness and improve the therapeutic efficiency of PI3K therapy (21). Similarly, our results demonstrated that TPP^+^-thiazole derivatives upregulated FoxO1 and downregulated Cyclin D1 protein levels. FoxO1 is reportedly involved in the mechanism of drug resistance, in which elevated ROS makes cancer cells more sensitive to chemotherapy. In addition, FoxOs can control ROS levels in response to chemotherapeutic agents (27). Because ROS levels have a significant effect FoxO family, the inhibitory effect of Mito-Fu L20 on FoxO1 suppression can be attributed to both inhibition of the PI3K/Akt pathway and ROS. Our findings suggest that mitochondrial dysfunction may be involved in the regulation of Cyclin D1 through an as-yet-unknown mechanism; therefore, further studies are needed to investigate the antiproliferative and proapoptotic effects of mitochondria-targeting drugs.

Intriguingly, we found changes in mitochondrial function induced by Mito-Fu L20 in several vital organs. The Mito-Fu family and its archetype, a cytochrome c oxidase inhibitor designed by Collman and Fu, have been reported to improve the quality and dynamics of mitochondria in the liver to correct the early stage of metabolic syndrome (4). After Mito-Fu L20 intervention in the present study, we found that the number of mitochondria elevated in the liver, suggesting that Mito-Fu L20 has a strong capacity for mitochondrial inhibition and specificity for tumor suppression. TSH is secreted by the adenohypophysis under the negative-feedback regulation of thyrotropin-releasing hormone from the hypothalamus and negative feedback from thyroid hormones, which reflects thyroid function and regulates the proliferation of thyroid cells (28). Tg is a glycoprotein secreted by thyroid follicular epithelial cells; it is therefore regarded as a tumor marker for differentiated thyroid cancer, including PTC and FTC (29). In the present study, TSH and Tg levels were significantly lower in peripheral blood of mice in the treatment groups than in the control group. These two indicators are clinically regarded as predictors of the proliferation or recurrence of differentiated thyroid cancer, suggesting that the influence of mitochondria in the hypothalamic-pituitary-thyroid axis affects endocrine function in the thyroid and metabolic function in tumors. Previous studies proposed that TSH stimulates hepatic CypD acetylation and plays an important role in mitochondrial stress in the liver (30). However, few studies have focused on changes in the associations of mitochondrial metabolic phenotypes related to thyroid function and hormones.

This study had some limitations. First, we have no direct evidence that Mito-Fu L20 affects mitochondrial complex IV, although our previous Western blotting analysis showed expression of complex IV bound to Mito-Fu (4), and our Seahorse experiments showed lower ATP production in treated groups. Furthermore, there is a lack of clarity regarding the regulation of mitochondrial respiration in PTC tumor progression through the PI3K/Akt/FoxO1/Cyclin D1 pathway. Further knockdown or overexpression studies may clarify the key regulators in this pathway.

In conclusion, changes in tumor metabolism, particularly in mitochondria respiration, resulted in considerable changes in tumor characteristics through the PI3K/Akt/FoxO1/Cyclin D1 pathway. Mito Fu, a novel TPP^+^-derived mitochondria-targeting inhibitor, showed a specific cytotoxicity toward tumor cells both *in vivo* and *in vitro.* The modulation of mitochondrial biogenesis by this drug resulted in substantial changes in the metabolic phenotype of PTC, which alleviated tumor migration and invasion, slowed cell cycle and tumor proliferation, promoted apoptosis, improved thyroid function, and inhibited tumor angiogenesis and LNM. These great antitumor effects suggest that members of the Mito-Fu family have potential as novel therapeutics agents and/or as components of combined adjuvant therapy for endocrine malignancies.

## Data Availability Statement

The raw data supporting the conclusions of this article will be made available by the authors, without undue reservation.

## Ethics Statement

The studies involving human participants were reviewed and approved by The Ethical Committee at Shanghai Jiao Tong University School of Medicine Affiliated Renji Hospital. The patients/participants provided their written informed consent to participate in this study. The animal study was reviewed and approved by the ethical committee at Shanghai Jiao Tong University School of Medicine Affiliated Renji Hospital.

## Author Contributions

BC *in vitro* and vivo experiments, data analysis, writing-original draft; SL design and synthesis of compounds, *in vitro* experiments, data analysis; XY and MF collection of clinical samples and information, related experiments and data analysis; YH *in vitro* experiments, writing-original draft; YS *in vivo* experiments; YX conceptualization, superior quality assessment, methodology, writing-review and editing; and LF conceived the project, designed research, provided study resources, analyzed data, and revised the manuscript. All authors contributed to the article and approved the submitted version.

## Funding

Medical and Industrial Cross Project of Shanghai Jiaotong University (YG2021QN41). National Natural Science Foundation of China (No.81900935). Shanghai Young Science and Technology Talents "Yang Fang Program" (No. 19YF1437600).

## Conflict of Interest

The authors declare that the research was conducted in the absence of any commercial or financial relationships that could be construed as a potential conflict of interest.

## Publisher’s Note

All claims expressed in this article are solely those of the authors and do not necessarily represent those of their affiliated organizations, or those of the publisher, the editors and the reviewers. Any product that may be evaluated in this article, or claim that may be made by its manufacturer, is not guaranteed or endorsed by the publisher.
