# Supplementary material for: Mitochondrial Respiration Inhibition Suppresses Papillary Thyroid Carcinoma Via PI3K/Akt/FoxO1/Cyclin D1 Pathway
Source: Front Oncol. 2022 Jul 5;12:900444. doi: 10.3389/fonc.2022.900444 (PMC9295996; doi:10.3389/fonc.2022.900444)
Supplement: Supplementary file 1 [file DataSheet_1.pdf]

Chinese version

Time of collection	姓名	ComplexIV IHC staining					
		high pos(%)	pos(%)	low pos(%)	neg(%)	H-score*100	H-score
2020-01-02 07:58	曹[redacted]	13	38	33	16	148	1.48
2020-01-03 13:01	龚[redacted]	15	38	30	17	151	1.51
2020-01-06 14:00	朱[redacted]	13	48	35	4	170	1.7
2020-01-07 12:08	吴[redacted]	13	45	30	12	159	1.59
2020-01-08 13:05	王[redacted]	15	35	30	20	145	1.45
2020-01-09 10:13	李[redacted]	17	30	25	28	136	1.36
2020-01-09 12:12	葛[redacted]	17	45	35	3	176	1.76
2020-01-09 12:26	王[redacted]	13	48	25	14	160	1.6
2020-01-09 18:06	张[redacted]	10	40	33	17	143	1.43
2020-01-10 12:28	卫[redacted]	15	30	33	22	138	1.38
2020-01-10 15:15	汪[redacted]	10	35	35	20	135	1.35
2020-01-10 15:17	田[redacted]	13	45	30	12	159	1.59
2020-01-13 10:04	顾[redacted]	13	43	28	16	153	1.53
2020-01-14 13:32	徐[redacted]	15	38	25	22	146	1.46
2020-01-14 16:22	蔡[redacted]	13	43	28	16	153	1.53
2020-01-16 11:23	李[redacted]	17	40	30	13	161	1.61
2020-01-16 14:13	汤[redacted]	13	35	30	22	139	1.39
2020-01-16 18:48	王[redacted]	10	35	25	30	125	1.25
2020-01-16 18:48	丁[redacted]	17	48	30	5	177	1.77
2020-01-17 12:24	刘[redacted]	10	38	25	27	131	1.31
2020-01-17 15:40	孙[redacted]	10	40	25	25	135	1.35
2020-01-21 09:52	张[redacted]	15	40	25	20	150	1.5
2020-02-03 11:52	陆[redacted]	13	38	30	19	145	1.45
2020-02-03 12:27	杜[redacted]	15	43	30	12	161	1.61
2020-02-04 10:53	沈[redacted]	13	30	33	24	132	1.32
2020-02-04 14:07	秦[redacted]	15	35	30	20	145	1.45
2020-02-06 12:10	张[redacted]	15	40	33	12	158	1.58
2020-02-06 12:11	李[redacted]	15	33	35	17	146	1.46
2020-02-06 14:33	肖[redacted]	10	38	25	27	131	1.31
2020-02-10 10:09	郁[redacted]	13	35	33	19	142	1.42
2020-02-13 09:38	薛[redacted]	10	43	28	19	144	1.44
2020-02-13 10:57	董[redacted]	10	40	28	22	138	1.38
2020-02-14 10:38	高[redacted]	10	40	35	15	145	1.45
2020-02-24 09:23	徐[redacted]	10	33	35	22	131	1.31
2020-02-25 12:36	林[redacted]	15	45	33	7	168	1.68
2020-03-05 09:46	韦[redacted]	10	45	30	15	150	1.5
2020-03-09 10:22	赵[redacted]	13	43	33	11	158	1.58
2020-03-13 10:17	金[redacted]	10	38	30	22	136	1.36
2020-03-16 12:03	董[redacted]	10	30	33	27	123	1.23
2020-03-17 09:39	于[redacted]	15	30	33	22	138	1.38
2020-03-18 11:02	倪[redacted]	15	48	33	4	174	1.74
2020-03-23 09:46	任[redacted]	15	30	35	20	140	1.4

2020-03-26 10:39	林[redacted]	17	40	28	15	159	1.59
2020-03-26 11:54	郑[redacted]	10	30	25	35	115	1.15
2020-03-26 13:35	张[redacted]	10	40	28	22	138	1.38
2020-03-26 14:42	唐[redacted]	17	30	30	23	141	1.41
2020-03-26 16:08	张[redacted]	17	38	35	10	162	1.62
2020-03-27 14:04	代[redacted]	17	38	25	20	152	1.52
2020-03-30 10:46	胡[redacted]	13	33	30	24	135	1.35
2020-03-30 13:38	叶[redacted]	13	43	25	19	150	1.5
2020-04-01 09:25	丁[redacted]	10	48	28	14	154	1.54
2020-04-02 11:16	刘[redacted]	13	30	35	22	134	1.34
2020-04-02 12:44	叶[redacted]	13	40	28	19	147	1.47
2020-04-03 14:06	何[redacted]	10	30	33	27	123	1.23
2020-04-07 09:39	秦[redacted]	10	33	33	24	129	1.29
2020-04-07 12:54	赵[redacted]	17	30	33	20	144	1.44
2020-04-07 15:27	余[redacted]	15	48	25	12	166	1.66
2020-04-09 15:34	赵[redacted]	13	30	35	22	134	1.34
2020-04-10 09:22	张[redacted]	15	38	30	17	151	1.51
2020-04-13 09:34	田[redacted]	10	35	30	25	130	1.3
2020-04-16 14:10	陆[redacted]	15	40	25	20	150	1.5
2020-04-17 13:23	须[redacted]	8	30	28	34	112	1.12
2020-04-20 09:14	张[redacted]	15	35	30	20	145	1.45
2020-04-20 13:21	徐[redacted]	8	43	28	21	138	1.38
2020-04-21 09:44	周[redacted]	15	35	25	25	140	1.4
2020-04-21 10:47	庄[redacted]	17	35	28	20	149	1.49
2020-04-21 13:55	罗[redacted]	15	40	25	20	150	1.5
2020-04-21 15:03	赵[redacted]	10	45	25	20	145	1.45
2020-04-21 17:27	董[redacted]	10	30	30	30	120	1.2
2020-04-23 12:17	陈[redacted]	17	38	30	15	157	1.57
2020-04-23 16:07	唐[redacted]	8	45	28	19	142	1.42
2020-04-24 15:26	陈[redacted]	17	40	33	10	164	1.64
2020-04-27 11:55	瞿[redacted]	15	38	25	22	146	1.46
2020-04-28 12:43	杨[redacted]	15	40	30	15	155	1.55
2020-04-28 13:11	马[redacted]	8	40	33	19	137	1.37
2020-04-28 13:57	黄[redacted]	15	40	30	15	155	1.55
2020-04-30 13:50	宋[redacted]	17	45	30	8	171	1.71
2020-04-30 14:06	刘[redacted]	17	43	35	5	172	1.72
2020-04-30 16:10	范[redacted]	17	45	28	10	169	1.69
2020-05-04 10:27	吴[redacted]	8	48	25	19	145	1.45
2020-05-04 13:56	何[redacted]	13	40	25	22	144	1.44
2020-05-04 16:35	马[redacted]	15	40	35	10	160	1.6
2020-05-05 12:33	杨[redacted]	10	35	30	25	130	1.3
2020-05-05 14:19	陈[redacted]	15	35	28	22	143	1.43
2020-05-05 15:30	俞[redacted]	13	38	25	24	140	1.4
2020-05-07 10:48	曹[redacted]	15	45	25	15	160	1.6
2020-05-07 13:41	李[redacted]	17	40	30	13	161	1.61

2020-05-07 16:39	王	[redacted]	13	40	35	12	154	1.54
2020-05-08 09:50	邱	[redacted]	13	40	25	22	144	1.44
2020-05-08 11:30	黄	[redacted]	17	33	35	15	152	1.52
2020-05-11 09:42	宋	[redacted]	10	45	30	15	150	1.5
2020-05-11 09:43	孙	[redacted]	10	35	28	27	128	1.28
2020-05-11 11:30	叶	[redacted]	17	48	28	7	175	1.75
2020-05-11 15:16	俞	[redacted]	17	45	30	8	171	1.71
2020-05-12 14:01	徐	[redacted]	15	40	30	15	155	1.55
2020-05-14 14:11	胡	[redacted]	15	43	35	7	166	1.66
2020-05-14 15:19	顾	[redacted]	15	30	25	30	130	1.3
2020-05-18 10:37	黎	[redacted]	15	38	33	14	154	1.54
2020-05-18 11:39	付	[redacted]	15	40	25	20	150	1.5
2020-05-19 09:38	余	[redacted]	8	43	30	19	140	1.4
2020-05-19 13:01	郁	[redacted]	15	30	35	20	140	1.4
2020-05-19 15:47	孙	[redacted]	15	38	33	14	154	1.54
2020-05-19 18:46	李	[redacted]	10	35	33	22	133	1.33
2020-05-21 10:07	邹	[redacted]	15	30	35	20	140	1.4
2020-05-21 16:21	闵	[redacted]	13	40	28	19	147	1.47
2020-05-21 17:47	章	[redacted]	15	40	35	10	160	1.6
2020-05-25 10:45	蔡	[redacted]	10	48	28	14	154	1.54
2020-05-25 12:48	马	[redacted]	17	35	30	18	151	1.51
2020-05-26 13:47	张	[redacted]	17	35	28	20	149	1.49
2020-05-26 15:46	闫	[redacted]	10	40	28	22	138	1.38
2020-05-26 17:12	金	[redacted]	17	35	30	18	151	1.51
2020-05-28 13:45	周	[redacted]	10	35	30	25	130	1.3
2020-05-28 15:44	夏	[redacted]	15	48	30	7	171	1.71
2020-05-28 15:45	林	[redacted]	8	45	35	12	149	1.49
2020-05-28 17:16	黄	[redacted]	13	43	28	16	153	1.53
2020-06-01 11:40	费	[redacted]	10	43	33	14	149	1.49
2020-06-01 13:26	陈	[redacted]	13	45	35	7	164	1.64
2020-06-02 14:03	刘	[redacted]	17	38	35	10	162	1.62
2020-06-02 17:35	陈	[redacted]	10	45	35	10	155	1.55
2020-06-04 10:18	钱	[redacted]	15	38	25	22	146	1.46
2020-06-04 12:09	陈	[redacted]	10	48	30	12	156	1.56
2020-06-04 13:56	顾	[redacted]	13	38	30	19	145	1.45
2020-06-04 15:36	陆	[redacted]	13	40	30	17	149	1.49
2020-06-05 14:25	王	[redacted]	15	33	35	17	146	1.46
2020-06-09 10:59	黄	[redacted]	10	35	28	27	128	1.28
2020-06-09 15:53	厉	[redacted]	10	43	30	17	146	1.46
2020-06-10 15:45	邵	[redacted]	10	35	30	25	130	1.3
2020-06-11 12:08	刘	[redacted]	10	30	25	35	115	1.15
2020-06-11 13:00	刘	[redacted]	10	38	30	22	136	1.36
2020-06-12 09:45	陈	[redacted]	15	35	25	25	140	1.4
2020-06-12 11:13	王	[redacted]	13	35	25	27	134	1.34
2020-06-12 13:41	谭	[redacted]	8	40	30	22	134	1.34

2020-06-15 10:55	张	[redacted]	8	38	25	29	125	1.25
2020-06-15 14:15	夏	[redacted]	8	35	33	24	127	1.27
2020-06-16 08:39	周	[redacted]	15	43	30	12	161	1.61
2020-06-16 10:29	谢	[redacted]	17	45	35	3	176	1.76
2020-06-17 15:58	陈	[redacted]	13	43	28	16	153	1.53
2020-06-17 16:32	姚	[redacted]	13	45	30	12	159	1.59
2020-06-18 09:50	陈	[redacted]	8	35	33	24	127	1.27
2020-06-18 09:57	瞿	[redacted]	17	40	30	13	161	1.61
2020-06-18 14:01	车	[redacted]	13	33	30	24	135	1.35
2020-06-22 09:38	陈	[redacted]	15	35	25	25	140	1.4
2020-06-22 10:49	孙	[redacted]	10	40	30	20	140	1.4
2020-06-22 15:29	李	[redacted]	10	33	25	32	121	1.21
2020-06-23 13:44	王	[redacted]	13	40	33	14	152	1.52
2020-06-26 15:29	丁	[redacted]	15	45	28	12	163	1.63
2020-06-29 10:38	张	[redacted]	10	45	28	17	148	1.48
2020-06-30 12:52	陈	[redacted]	8	38	25	29	125	1.25
2020-06-30 18:25	沈	[redacted]	15	48	35	2	176	1.76
2020-07-01 09:18	洪	[redacted]	15	43	28	14	159	1.59
2020-07-02 10:09	郝	[redacted]	8	43	25	24	135	1.35
2020-07-02 11:51	万	[redacted]	17	43	35	5	172	1.72
2020-07-02 15:11	岳	[redacted]	10	38	25	27	131	1.31
2020-07-02 17:20	郑	[redacted]	17	35	28	20	149	1.49
2020-07-06 10:29	曹	[redacted]	13	43	30	14	155	1.55
2020-07-06 12:00	向	[redacted]	15	33	25	27	136	1.36
2020-07-07 12:59	万	[redacted]	13	33	33	21	138	1.38
2020-07-07 15:54	储	[redacted]	8	35	30	27	124	1.24
2020-07-09 09:48	王	[redacted]	17	35	33	15	154	1.54
2020-07-09 13:22	刘	[redacted]	15	48	30	7	171	1.71
2020-07-09 13:23	孙	[redacted]	15	38	33	14	154	1.54
2020-07-10 14:37	蔡	[redacted]	15	38	30	17	151	1.51
2020-07-13 13:21	骆	[redacted]	13	45	35	7	164	1.64
2020-07-13 14:05	余	[redacted]	10	40	30	20	140	1.4
2020-07-14 15:27	虞	[redacted]	10	30	35	25	125	1.25
2020-07-15 12:00	宗	[redacted]	17	30	30	23	141	1.41
2020-07-15 13:09	王	[redacted]	10	45	33	12	153	1.53
2020-07-16 13:01	张	[redacted]	15	38	30	17	151	1.51
2020-07-16 16:56	徐	[redacted]	8	33	30	29	120	1.2
2020-07-17 09:39	庄	[redacted]	10	48	25	17	151	1.51
2020-07-17 12:21	刘	[redacted]	13	40	33	14	152	1.52
2020-07-17 13:30	徐	[redacted]	17	45	33	5	174	1.74
2020-07-20 13:30	陈	[redacted]	10	40	33	17	143	1.43
2020-07-20 19:24	吴	[redacted]	17	33	28	22	145	1.45
2020-07-21 09:24	顾	[redacted]	13	30	35	22	134	1.34
2020-07-21 15:52	陈	[redacted]	15	43	33	9	164	1.64
2020-07-21 17:33	张	[redacted]	10	30	30	30	120	1.2

2020-07-22 15:57	史	[redacted]	10	45	25	20	145	1.45
2020-07-23 12:37	孙	[redacted]	13	40	35	12	154	1.54
2020-07-24 11:58	陈	[redacted]	20	45	30	5	180	1.8
2020-07-24 14:20	于	[redacted]	8	30	30	32	114	1.14
2020-07-27 16:08	朱	[redacted]	13	43	28	16	153	1.53
2020-07-28 12:17	徐	[redacted]	13	30	28	29	127	1.27
2020-07-28 15:59	章	[redacted]	8	38	30	24	130	1.3
2020-07-28 16:49	张	[redacted]	15	45	35	5	170	1.7
2020-07-29 12:18	章	[redacted]	13	35	25	27	134	1.34
2020-07-29 15:59	王	[redacted]	13	33	25	29	130	1.3
2020-07-30 12:50	居	[redacted]	10	45	30	15	150	1.5
2020-07-31 15:56	谭	[redacted]	17	40	25	18	156	1.56
2020-08-03 09:52	浦	[redacted]	10	45	28	17	148	1.48
2020-08-03 10:14	谢	[redacted]	8	33	28	31	118	1.18
2020-08-03 11:18	戴	[redacted]	10	45	28	17	148	1.48
2020-08-04 10:21	王	[redacted]	15	48	25	12	166	1.66
2020-08-05 10:07	马	[redacted]	8	38	33	21	133	1.33
2020-08-05 10:30	沈	[redacted]	10	43	33	14	149	1.49
2020-08-06 10:46	唐	[redacted]	10	48	33	9	159	1.59
2020-08-10 10:47	李	[redacted]	10	35	30	25	130	1.3
2020-08-10 13:37	侯	[redacted]	15	48	28	9	169	1.69
2020-08-11 15:23	季	[redacted]	20	33	35	12	161	1.61
2020-08-12 09:33	赵	[redacted]	10	45	25	20	145	1.45
2020-08-13 16:00	何	[redacted]	10	30	28	32	118	1.18
2020-08-14 12:27	李	[redacted]	8	45	33	14	147	1.47
2020-08-14 14:55	李	[redacted]	10	48	33	9	159	1.59
2020-08-14 17:10	周	[redacted]	17	45	35	3	176	1.76
2020-08-14 18:07	陈	[redacted]	8	30	30	32	114	1.14
2020-08-17 10:44	万	[redacted]	15	35	35	15	150	1.5
2020-08-17 12:02	葛	[redacted]	15	45	28	12	163	1.63
2020-08-17 13:03	刘	[redacted]	10	33	30	27	126	1.26
2020-08-18 11:20	王	[redacted]	15	38	28	19	149	1.49
2020-08-18 11:56	周	[redacted]	10	40	28	22	138	1.38
2020-08-20 15:21	颜	[redacted]	20	38	30	12	166	1.66
2020-08-21 10:41	徐	[redacted]	20	40	33	7	173	1.73
2020-08-25 15:52	周	[redacted]	10	43	28	19	144	1.44
2020-08-26 14:22	周	[redacted]	15	35	28	22	143	1.43
2020-08-27 13:22	苏	[redacted]	17	40	25	18	156	1.56
2020-08-28 09:46	钟	[redacted]	17	40	35	8	166	1.66
2020-08-28 09:56	周	[redacted]	10	38	33	19	139	1.39
2020-08-28 10:53	李	[redacted]	15	33	28	24	139	1.39
2020-08-31 11:22	吕	[redacted]	10	43	30	17	146	1.46
2020-08-31 15:03	卫	[redacted]	10	45	30	15	150	1.5
2020-09-01 16:19	桑	[redacted]	15	38	28	19	149	1.49
2020-09-02 09:53	祝	[redacted]	10	38	30	22	136	1.36

2020-09-07 13:50	邱	[redacted]	10	35	35	20	135	1.35
2020-09-07 13:51	王		15	45	25	15	160	1.6
2020-09-08	程		15	45	28	12	163	1.63
2020-09-09 11:01	邱		15	43	33	9	164	1.64
2020-09-10 10:07	兰		15	45	33	7	168	1.68
2020-09-11 11:04	胡		15	38	30	17	151	1.51
2020-09-11 14:20	何		15	30	28	27	133	1.33
2020-09-14 09:43	王		15	48	30	7	171	1.71
2020-09-14 11:30	李		20	38	28	14	164	1.64
2020-09-15 11:51	杨		13	48	30	9	165	1.65
2020-09-15 14:04	陈		15	38	30	17	151	1.51
2020-09-15 15:37	江		13	40	30	17	149	1.49
2020-09-16	徐		10	40	28	22	138	1.38
2020-09-16 09:20	杨		8	38	35	19	135	1.35
2020-09-16 19:35	吴		15	33	30	22	141	1.41
2020-09-18 13:05	江		10	35	28	27	128	1.28
2020-09-22 09:36	范		15	45	30	10	165	1.65
2020-09-22 09:37	应		13	45	25	17	154	1.54
2020-09-22 11:36	黄		15	45	30	10	165	1.65
2020-09-22 12:47	花		15	38	30	17	151	1.51
2020-09-22 15:49	孟		15	35	25	25	140	1.4
2020-09-24 13:02	徐		17	38	25	20	152	1.52
2020-09-25 10:41	宋		17	45	33	5	174	1.74
2020-10-12 11:54	祝		17	40	28	15	159	1.59
2020-10-13 15:51	李		10	33	33	24	129	1.29
2020-10-13 15:52	朱		20	43	25	12	171	1.71
2020-10-14 09:33	高		20	45	25	10	175	1.75
2020-10-14 14:27	刘		13	43	30	14	155	1.55
2020-10-20 10:06	宋		15	43	33	9	164	1.64
2020-10-20 13:28	张		8	33	30	29	120	1.2
2020-10-21 14:59	赵		15	43	35	7	166	1.66
2020-10-22 11:43	陈		15	38	25	22	146	1.46
2020-10-26 10:15	夏		8	45	25	22	139	1.39
2020-10-30 13:41	许		15	48	30	7	171	1.71
2020-10-30 14:57	钱		15	43	30	12	161	1.61
2020-11-02 09:11	应		13	40	25	22	144	1.44
2020-11-02 14:29	刘		20	38	35	7	171	1.71
2020-11-04 14:00	沈		17	40	25	18	156	1.56
2020-11-06 12:40	丁		10	45	30	15	150	1.5
2020-11-06 13:50	田		10	43	35	12	151	1.51
2020-11-09	代		13	40	28	19	147	1.47
2020-11-09	杨		15	33	30	22	141	1.41
2020-11-09 13:41	俞		10	45	30	15	150	1.5
2020-11-09 15:31	秦		15	35	30	20	145	1.45
2020-11-10 13:24	胡		15	40	28	17	153	1.53

2020-11-11 11:50	袁	[redacted]	10	30	33	27	123	1.23
2020-11-13 14:51	谭	[redacted]	20	43	33	4	179	1.79
2020-11-13 17:33	陆	[redacted]	10	40	28	22	138	1.38
2020-11-13 19:28	钟	[redacted]	17	48	33	2	180	1.8
2020-11-16	张	[redacted]	10	48	35	7	161	1.61
2020-11-16 16:02	刘	[redacted]	10	38	30	22	136	1.36
2020-11-17 09:25	冯	[redacted]	8	30	33	29	117	1.17
2020-11-17 13:20	许	[redacted]	13	33	30	24	135	1.35
2020-11-17 17:55	郑	[redacted]	10	45	35	10	155	1.55
2020-11-18 13:55	张	[redacted]	15	43	30	12	161	1.61
2020-11-19 11:13	马	[redacted]	20	40	28	12	168	1.68
2020-11-23	杜	[redacted]	15	40	30	15	155	1.55
2020-11-23 09:41	倪	[redacted]	13	35	33	19	142	1.42
2020-11-23 14:35	成	[redacted]	13	30	33	24	132	1.32
2020-11-23 15:30	施	[redacted]	17	38	28	17	155	1.55
2020-11-23 16:16	周	[redacted]	17	33	35	15	152	1.52
2020-11-23 16:54	蒋	[redacted]	13	30	25	32	124	1.24
2020-11-24 10:41	沈	[redacted]	13	30	33	24	132	1.32
2020-11-24 15:26	张	[redacted]	15	35	30	20	145	1.45
2020-11-24 16:31	丁	[redacted]	17	48	25	10	172	1.72
2020-11-24 18:28	徐	[redacted]	13	38	30	19	145	1.45
2020-11-25 10:35	颜	[redacted]	10	48	25	17	151	1.51
2020-11-27	朱	[redacted]	8	38	35	19	135	1.35
2020-11-27 10:03	李	[redacted]	13	43	28	16	153	1.53
2020-11-27 11:36	吴	[redacted]	10	43	25	22	141	1.41
2020-11-30 13:32	赵	[redacted]	15	40	35	10	160	1.6
2020-12-01 10:27	张	[redacted]	13	45	28	14	157	1.57
2020-12-01 12:38	李	[redacted]	15	45	33	7	168	1.68
2020-12-01 16:41	陈	[redacted]	10	43	28	19	144	1.44
2020-12-02 09:35	康	[redacted]	13	35	30	22	139	1.39
2020-12-02 10:20	杨	[redacted]	15	45	30	10	165	1.65
2020-12-04 09:52	王	[redacted]	8	35	30	27	124	1.24
2020-12-07 12:24	郑	[redacted]	20	35	30	15	160	1.6
2020-12-07 14:01	陈	[redacted]	10	30	30	30	120	1.2
2020-12-07 17:43	吴	[redacted]	10	33	28	29	124	1.24
2020-12-08 14:17	潘	[redacted]	13	45	35	7	164	1.64
2020-12-08 17:35	王	[redacted]	8	35	33	24	127	1.27
2020-12-09 14:01	何	[redacted]	15	48	30	7	171	1.71
2020-12-11	王	[redacted]	10	35	28	27	128	1.28
2020-12-14 15:30	黄	[redacted]	15	45	28	12	163	1.63
2020-12-15 16:24	王	[redacted]	10	35	30	25	130	1.3
2020-12-16 11:02	翟	[redacted]	8	33	30	29	120	1.2
2020-12-18	任	[redacted]	10	33	35	22	131	1.31
2020-12-21 10:53	汤	[redacted]	15	38	28	19	149	1.49
2020-12-22 14:53	王	[redacted]	8	43	33	16	143	1.43

2020-12-23	邬[redacted]	15	43	28	14	159	1.59
2020-12-23 11:19	徐[redacted]	10	33	35	22	131	1.31
2020-12-25 14:25	张[redacted]	8	48	30	14	150	1.5
2020-12-25 16:30	姚[redacted]	13	40	30	17	149	1.49
2020-12-28 11:43	王[redacted]	15	45	30	10	165	1.65
2020-12-30	唐[redacted]	15	35	30	20	145	1.45
2020-12-30 12:59	姜[redacted]	10	35	28	27	128	1.28
					median	149	1.49
					quartile (25%)	136.5	1.365
					quartile (75%)	159	1.59

English version

Time of collection	Family Name	ComplexIV IHC staining					
		high pos(%)	pos(%)	low pos(%)	neg(%)	H-score*100	H-score
2020-01-02 07:58	Cao	13	38	33	16	148	1.48
2020-01-03 13:01	Gong	15	38	30	17	151	1.51
2020-01-06 14:00	Zhu	13	48	35	4	170	1.7
2020-01-07 12:08	Wu	13	45	30	12	159	1.59
2020-01-08 13:05	Wang	15	35	30	20	145	1.45
2020-01-09 10:13	Li	17	30	25	28	136	1.36
2020-01-09 12:12	Ge	17	45	35	3	176	1.76
2020-01-09 12:26	Wang	13	48	25	14	160	1.6
2020-01-09 18:06	Zhang	10	40	33	17	143	1.43
2020-01-10 12:28	Wei	15	30	33	22	138	1.38
2020-01-10 15:15	Wang	10	35	35	20	135	1.35
2020-01-10 15:17	Tian	13	45	30	12	159	1.59
2020-01-13 10:04	Gu	13	43	28	16	153	1.53
2020-01-14 13:32	Xu	15	38	25	22	146	1.46
2020-01-14 16:22	Cai	13	43	28	16	153	1.53
2020-01-16 11:23	Li	17	40	30	13	161	1.61
2020-01-16 14:13	Tang	13	35	30	22	139	1.39
2020-01-16 18:48	Wang	10	35	25	30	125	1.25
2020-01-16 18:48	Ding	17	48	30	5	177	1.77
2020-01-17 12:24	Liu	10	38	25	27	131	1.31
2020-01-17 15:40	Sun	10	40	25	25	135	1.35
2020-01-21 09:52	Zhang	15	40	25	20	150	1.5
2020-02-03 11:52	Lu	13	38	30	19	145	1.45
2020-02-03 12:27	Du	15	43	30	12	161	1.61
2020-02-04 10:53	Shen	13	30	33	24	132	1.32
2020-02-04 14:07	Qin	15	35	30	20	145	1.45
2020-02-06 12:10	Zhang	15	40	33	12	158	1.58
2020-02-06 12:11	Li	15	33	35	17	146	1.46
2020-02-06 14:33	Xiao	10	38	25	27	131	1.31
2020-02-10 10:09	Yu	13	35	33	19	142	1.42
2020-02-13 09:38	Xue	10	43	28	19	144	1.44
2020-02-13 10:57	Dong	10	40	28	22	138	1.38
2020-02-14 10:38	Gao	10	40	35	15	145	1.45
2020-02-24 09:23	Xu	10	33	35	22	131	1.31
2020-02-25 12:36	Lin	15	45	33	7	168	1.68
2020-03-05 09:46	Wei	10	45	30	15	150	1.5
2020-03-09 10:22	Zhao	13	43	33	11	158	1.58
2020-03-13 10:17	Jin	10	38	30	22	136	1.36
2020-03-16 12:03	Dong	10	30	33	27	123	1.23
2020-03-17 09:39	Yu	15	30	33	22	138	1.38
2020-03-18 11:02	Ni	15	48	33	4	174	1.74
2020-03-23 09:46	Ren	15	30	35	20	140	1.4

2020-03-26 10:39	Lin	17	40	28	15	159	1.59
2020-03-26 11:54	Zheng	10	30	25	35	115	1.15
2020-03-26 13:35	Zhang	10	40	28	22	138	1.38
2020-03-26 14:42	Tang	17	30	30	23	141	1.41
2020-03-26 16:08	Zhang	17	38	35	10	162	1.62
2020-03-27 14:04	Dai	17	38	25	20	152	1.52
2020-03-30 10:46	Hu	13	33	30	24	135	1.35
2020-03-30 13:38	Ye	13	43	25	19	150	1.5
2020-04-01 09:25	Ding	10	48	28	14	154	1.54
2020-04-02 11:16	Liu	13	30	35	22	134	1.34
2020-04-02 12:44	Ye	13	40	28	19	147	1.47
2020-04-03 14:06	He	10	30	33	27	123	1.23
2020-04-07 09:39	Qin	10	33	33	24	129	1.29
2020-04-07 12:54	Zhao	17	30	33	20	144	1.44
2020-04-07 15:27	Yu	15	48	25	12	166	1.66
2020-04-09 15:34	Zhao	13	30	35	22	134	1.34
2020-04-10 09:22	Zhang	15	38	30	17	151	1.51
2020-04-13 09:34	Tian	10	35	30	25	130	1.3
2020-04-16 14:10	Lu	15	40	25	20	150	1.5
2020-04-17 13:23	Xu	8	30	28	34	112	1.12
2020-04-20 09:14	Zhang	15	35	30	20	145	1.45
2020-04-20 13:21	Xu	8	43	28	21	138	1.38
2020-04-21 09:44	Zhou	15	35	25	25	140	1.4
2020-04-21 10:47	Zhuang	17	35	28	20	149	1.49
2020-04-21 13:55	Luo	15	40	25	20	150	1.5
2020-04-21 15:03	Zhao	10	45	25	20	145	1.45
2020-04-21 17:27	Dong	10	30	30	30	120	1.2
2020-04-23 12:17	Chen	17	38	30	15	157	1.57
2020-04-23 16:07	Tang	8	45	28	19	142	1.42
2020-04-24 15:26	Chen	17	40	33	10	164	1.64
2020-04-27 11:55	Qu	15	38	25	22	146	1.46
2020-04-28 12:43	Yang	15	40	30	15	155	1.55
2020-04-28 13:11	Ma	8	40	33	19	137	1.37
2020-04-28 13:57	Huang	15	40	30	15	155	1.55
2020-04-30 13:50	Song	17	45	30	8	171	1.71
2020-04-30 14:06	Liu	17	43	35	5	172	1.72
2020-04-30 16:10	Fan	17	45	28	10	169	1.69
2020-05-04 10:27	Wu	8	48	25	19	145	1.45
2020-05-04 13:56	He	13	40	25	22	144	1.44
2020-05-04 16:35	Ma	15	40	35	10	160	1.6
2020-05-05 12:33	Yang	10	35	30	25	130	1.3
2020-05-05 14:19	Chen	15	35	28	22	143	1.43
2020-05-05 15:30	Yu	13	38	25	24	140	1.4
2020-05-07 10:48	Cao	15	45	25	15	160	1.6
2020-05-07 13:41	Li	17	40	30	13	161	1.61

2020-05-07 16:39	Wang	13	40	35	12	154	1.54
2020-05-08 09:50	Qiu	13	40	25	22	144	1.44
2020-05-08 11:30	Huang	17	33	35	15	152	1.52
2020-05-11 09:42	Song	10	45	30	15	150	1.5
2020-05-11 09:43	Sun	10	35	28	27	128	1.28
2020-05-11 11:30	Ye	17	48	28	7	175	1.75
2020-05-11 15:16	Yu	17	45	30	8	171	1.71
2020-05-12 14:01	Xu	15	40	30	15	155	1.55
2020-05-14 14:11	Hu	15	43	35	7	166	1.66
2020-05-14 15:19	Gu	15	30	25	30	130	1.3
2020-05-18 10:37	Li	15	38	33	14	154	1.54
2020-05-18 11:39	Fu	15	40	25	20	150	1.5
2020-05-19 09:38	Yu	8	43	30	19	140	1.4
2020-05-19 13:01	Yu	15	30	35	20	140	1.4
2020-05-19 15:47	Sun	15	38	33	14	154	1.54
2020-05-19 18:46	Li	10	35	33	22	133	1.33
2020-05-21 10:07	Zou	15	30	35	20	140	1.4
2020-05-21 16:21	Min	13	40	28	19	147	1.47
2020-05-21 17:47	Zhang	15	40	35	10	160	1.6
2020-05-25 10:45	Cai	10	48	28	14	154	1.54
2020-05-25 12:48	Ma	17	35	30	18	151	1.51
2020-05-26 13:47	Zhang	17	35	28	20	149	1.49
2020-05-26 15:46	Yan	10	40	28	22	138	1.38
2020-05-26 17:12	Jin	17	35	30	18	151	1.51
2020-05-28 13:45	Zhou	10	35	30	25	130	1.3
2020-05-28 15:44	Xia	15	48	30	7	171	1.71
2020-05-28 15:45	Lin	8	45	35	12	149	1.49
2020-05-28 17:16	Huang	13	43	28	16	153	1.53
2020-06-01 11:40	Fei	10	43	33	14	149	1.49
2020-06-01 13:26	Chen	13	45	35	7	164	1.64
2020-06-02 14:03	Liu	17	38	35	10	162	1.62
2020-06-02 17:35	Chen	10	45	35	10	155	1.55
2020-06-04 10:18	Qian	15	38	25	22	146	1.46
2020-06-04 12:09	Chen	10	48	30	12	156	1.56
2020-06-04 13:56	Gu	13	38	30	19	145	1.45
2020-06-04 15:36	Lu	13	40	30	17	149	1.49
2020-06-05 14:25	Wang	15	33	35	17	146	1.46
2020-06-09 10:59	Huang	10	35	28	27	128	1.28
2020-06-09 15:53	Li	10	43	30	17	146	1.46
2020-06-10 15:45	Shao	10	35	30	25	130	1.3
2020-06-11 12:08	Liu	10	30	25	35	115	1.15
2020-06-11 13:00	Liu	10	38	30	22	136	1.36
2020-06-12 09:45	Chen	15	35	25	25	140	1.4
2020-06-12 11:13	Wang	13	35	25	27	134	1.34
2020-06-12 13:41	Tan	8	40	30	22	134	1.34

2020-06-15 10:55	Zhang	8	38	25	29	125	1.25
2020-06-15 14:15	Xia	8	35	33	24	127	1.27
2020-06-16 08:39	Zhou	15	43	30	12	161	1.61
2020-06-16 10:29	Xie	17	45	35	3	176	1.76
2020-06-17 15:58	Chen	13	43	28	16	153	1.53
2020-06-17 16:32	Yao	13	45	30	12	159	1.59
2020-06-18 09:50	Chen	8	35	33	24	127	1.27
2020-06-18 09:57	Qu	17	40	30	13	161	1.61
2020-06-18 14:01	Che	13	33	30	24	135	1.35
2020-06-22 09:38	Chen	15	35	25	25	140	1.4
2020-06-22 10:49	Sun	10	40	30	20	140	1.4
2020-06-22 15:29	Li	10	33	25	32	121	1.21
2020-06-23 13:44	Wang	13	40	33	14	152	1.52
2020-06-26 15:29	Ding	15	45	28	12	163	1.63
2020-06-29 10:38	Zhang	10	45	28	17	148	1.48
2020-06-30 12:52	Chen	8	38	25	29	125	1.25
2020-06-30 18:25	Shen	15	48	35	2	176	1.76
2020-07-01 09:18	Hong	15	43	28	14	159	1.59
2020-07-02 10:09	Hao	8	43	25	24	135	1.35
2020-07-02 11:51	Wan	17	43	35	5	172	1.72
2020-07-02 15:11	Yue	10	38	25	27	131	1.31
2020-07-02 17:20	Zheng	17	35	28	20	149	1.49
2020-07-06 10:29	Cao	13	43	30	14	155	1.55
2020-07-06 12:00	Xiang	15	33	25	27	136	1.36
2020-07-07 12:59	Wan	13	33	33	21	138	1.38
2020-07-07 15:54	Chu	8	35	30	27	124	1.24
2020-07-09 09:48	Wang	17	35	33	15	154	1.54
2020-07-09 13:22	Liu	15	48	30	7	171	1.71
2020-07-09 13:23	Sun	15	38	33	14	154	1.54
2020-07-10 14:37	Cai	15	38	30	17	151	1.51
2020-07-13 13:21	Luo	13	45	35	7	164	1.64
2020-07-13 14:05	Yu	10	40	30	20	140	1.4
2020-07-14 15:27	Yu	10	30	35	25	125	1.25
2020-07-15 12:00	Zong	17	30	30	23	141	1.41
2020-07-15 13:09	Wang	10	45	33	12	153	1.53
2020-07-16 13:01	Zhang	15	38	30	17	151	1.51
2020-07-16 16:56	Xu	8	33	30	29	120	1.2
2020-07-17 09:39	Zhuang	10	48	25	17	151	1.51
2020-07-17 12:21	Liu	13	40	33	14	152	1.52
2020-07-17 13:30	Xu	17	45	33	5	174	1.74
2020-07-20 13:30	Chen	10	40	33	17	143	1.43
2020-07-20 19:24	Wu	17	33	28	22	145	1.45
2020-07-21 09:24	Gu	13	30	35	22	134	1.34
2020-07-21 15:52	Chen	15	43	33	9	164	1.64
2020-07-21 17:33	Zhang	10	30	30	30	120	1.2

2020-07-22 15:57	Shi	10	45	25	20	145	1.45
2020-07-23 12:37	Sun	13	40	35	12	154	1.54
2020-07-24 11:58	Chen	20	45	30	5	180	1.8
2020-07-24 14:20	Yu	8	30	30	32	114	1.14
2020-07-27 16:08	Zhu	13	43	28	16	153	1.53
2020-07-28 12:17	Xu	13	30	28	29	127	1.27
2020-07-28 15:59	Zhang	8	38	30	24	130	1.3
2020-07-28 16:49	Zhang	15	45	35	5	170	1.7
2020-07-29 12:18	Zhang	13	35	25	27	134	1.34
2020-07-29 15:59	Wang	13	33	25	29	130	1.3
2020-07-30 12:50	Ju	10	45	30	15	150	1.5
2020-07-31 15:56	Tan	17	40	25	18	156	1.56
2020-08-03 09:52	Pu	10	45	28	17	148	1.48
2020-08-03 10:14	Xie	8	33	28	31	118	1.18
2020-08-03 11:18	Dai	10	45	28	17	148	1.48
2020-08-04 10:21	Wang	15	48	25	12	166	1.66
2020-08-05 10:07	Ma	8	38	33	21	133	1.33
2020-08-05 10:30	Shen	10	43	33	14	149	1.49
2020-08-06 10:46	Tang	10	48	33	9	159	1.59
2020-08-10 10:47	Li	10	35	30	25	130	1.3
2020-08-10 13:37	Hou	15	48	28	9	169	1.69
2020-08-11 15:23	Ji	20	33	35	12	161	1.61
2020-08-12 09:33	Zhao	10	45	25	20	145	1.45
2020-08-13 16:00	He	10	30	28	32	118	1.18
2020-08-14 12:27	Li	8	45	33	14	147	1.47
2020-08-14 14:55	Li	10	48	33	9	159	1.59
2020-08-14 17:10	Zhou	17	45	35	3	176	1.76
2020-08-14 18:07	Chen	8	30	30	32	114	1.14
2020-08-17 10:44	Wan	15	35	35	15	150	1.5
2020-08-17 12:02	Ge	15	45	28	12	163	1.63
2020-08-17 13:03	Liu	10	33	30	27	126	1.26
2020-08-18 11:20	Wang	15	38	28	19	149	1.49
2020-08-18 11:56	Zhou	10	40	28	22	138	1.38
2020-08-20 15:21	Yan	20	38	30	12	166	1.66
2020-08-21 10:41	Xu	20	40	33	7	173	1.73
2020-08-25 15:52	Zhou	10	43	28	19	144	1.44
2020-08-26 14:22	Zhou	15	35	28	22	143	1.43
2020-08-27 13:22	Su	17	40	25	18	156	1.56
2020-08-28 09:46	Zhong	17	40	35	8	166	1.66
2020-08-28 09:56	Zhou	10	38	33	19	139	1.39
2020-08-28 10:53	Li	15	33	28	24	139	1.39
2020-08-31 11:22	Lv	10	43	30	17	146	1.46
2020-08-31 15:03	Wei	10	45	30	15	150	1.5
2020-09-01 16:19	Sang	15	38	28	19	149	1.49
2020-09-02 09:53	Zhu	10	38	30	22	136	1.36

2020-09-07 13:50	Qiu	10	35	35	20	135	1.35
2020-09-07 13:51	Wang	15	45	25	15	160	1.6
2020-09-08	Cheng	15	45	28	12	163	1.63
2020-09-09 11:01	Qiu	15	43	33	9	164	1.64
2020-09-10 10:07	Lan	15	45	33	7	168	1.68
2020-09-11 11:04	Hu	15	38	30	17	151	1.51
2020-09-11 14:20	He	15	30	28	27	133	1.33
2020-09-14 09:43	Wang	15	48	30	7	171	1.71
2020-09-14 11:30	Li	20	38	28	14	164	1.64
2020-09-15 11:51	Yang	13	48	30	9	165	1.65
2020-09-15 14:04	Chen	15	38	30	17	151	1.51
2020-09-15 15:37	Jiang	13	40	30	17	149	1.49
2020-09-16	Xu	10	40	28	22	138	1.38
2020-09-16 09:20	Yang	8	38	35	19	135	1.35
2020-09-16 19:35	Wu	15	33	30	22	141	1.41
2020-09-18 13:05	Jiang	10	35	28	27	128	1.28
2020-09-22 09:36	Fan	15	45	30	10	165	1.65
2020-09-22 09:37	Ying	13	45	25	17	154	1.54
2020-09-22 11:36	Huang	15	45	30	10	165	1.65
2020-09-22 12:47	Hua	15	38	30	17	151	1.51
2020-09-22 15:49	Meng	15	35	25	25	140	1.4
2020-09-24 13:02	Xu	17	38	25	20	152	1.52
2020-09-25 10:41	Song	17	45	33	5	174	1.74
2020-10-12 11:54	Zhu	17	40	28	15	159	1.59
2020-10-13 15:51	Li	10	33	33	24	129	1.29
2020-10-13 15:52	Zhu	20	43	25	12	171	1.71
2020-10-14 09:33	Gao	20	45	25	10	175	1.75
2020-10-14 14:27	Liu	13	43	30	14	155	1.55
2020-10-20 10:06	Song	15	43	33	9	164	1.64
2020-10-20 13:28	Zhang	8	33	30	29	120	1.2
2020-10-21 14:59	Zhao	15	43	35	7	166	1.66
2020-10-22 11:43	Chen	15	38	25	22	146	1.46
2020-10-26 10:15	Xia	8	45	25	22	139	1.39
2020-10-30 13:41	Xu	15	48	30	7	171	1.71
2020-10-30 14:57	Qian	15	43	30	12	161	1.61
2020-11-02 09:11	Ying	13	40	25	22	144	1.44
2020-11-02 14:29	Liu	20	38	35	7	171	1.71
2020-11-04 14:00	Shen	17	40	25	18	156	1.56
2020-11-06 12:40	Ding	10	45	30	15	150	1.5
2020-11-06 13:50	Tian	10	43	35	12	151	1.51
2020-11-09	Dai	13	40	28	19	147	1.47
2020-11-09	Yang	15	33	30	22	141	1.41
2020-11-09 13:41	Yu	10	45	30	15	150	1.5
2020-11-09 15:31	Qin	15	35	30	20	145	1.45
2020-11-10 13:24	Hu	15	40	28	17	153	1.53

2020-11-11 11:50	Yuan	10	30	33	27	123	1.23
2020-11-13 14:51	Tan	20	43	33	4	179	1.79
2020-11-13 17:33	Lu	10	40	28	22	138	1.38
2020-11-13 19:28	Zhong	17	48	33	2	180	1.8
2020-11-16	Zhang	10	48	35	7	161	1.61
2020-11-16 16:02	Liu	10	38	30	22	136	1.36
2020-11-17 09:25	Feng	8	30	33	29	117	1.17
2020-11-17 13:20	Xu	13	33	30	24	135	1.35
2020-11-17 17:55	Zheng	10	45	35	10	155	1.55
2020-11-18 13:55	Zhang	15	43	30	12	161	1.61
2020-11-19 11:13	Ma	20	40	28	12	168	1.68
2020-11-23	Du	15	40	30	15	155	1.55
2020-11-23 09:41	Ni	13	35	33	19	142	1.42
2020-11-23 14:35	Cheng	13	30	33	24	132	1.32
2020-11-23 15:30	Shi	17	38	28	17	155	1.55
2020-11-23 16:16	Zhou	17	33	35	15	152	1.52
2020-11-23 16:54	Jiang	13	30	25	32	124	1.24
2020-11-24 10:41	Shen	13	30	33	24	132	1.32
2020-11-24 15:26	Zhang	15	35	30	20	145	1.45
2020-11-24 16:31	Ding	17	48	25	10	172	1.72
2020-11-24 18:28	Xu	13	38	30	19	145	1.45
2020-11-25 10:35	Yan	10	48	25	17	151	1.51
2020-11-27	Zhu	8	38	35	19	135	1.35
2020-11-27 10:03	Li	13	43	28	16	153	1.53
2020-11-27 11:36	Wu	10	43	25	22	141	1.41
2020-11-30 13:32	Zhao	15	40	35	10	160	1.6
2020-12-01 10:27	Zhang	13	45	28	14	157	1.57
2020-12-01 12:38	Li	15	45	33	7	168	1.68
2020-12-01 16:41	Chen	10	43	28	19	144	1.44
2020-12-02 09:35	Kang	13	35	30	22	139	1.39
2020-12-02 10:20	Yang	15	45	30	10	165	1.65
2020-12-04 09:52	Wang	8	35	30	27	124	1.24
2020-12-07 12:24	Zheng	20	35	30	15	160	1.6
2020-12-07 14:01	Chen	10	30	30	30	120	1.2
2020-12-07 17:43	Wu	10	33	28	29	124	1.24
2020-12-08 14:17	Pan	13	45	35	7	164	1.64
2020-12-08 17:35	Wang	8	35	33	24	127	1.27
2020-12-09 14:01	He	15	48	30	7	171	1.71
2020-12-11	Wang	10	35	28	27	128	1.28
2020-12-14 15:30	Huang	15	45	28	12	163	1.63
2020-12-15 16:24	Wang	10	35	30	25	130	1.3
2020-12-16 11:02	Zhai	8	33	30	29	120	1.2
2020-12-18	Ren	10	33	35	22	131	1.31
2020-12-21 10:53	TAng	15	38	28	19	149	1.49
2020-12-22 14:53	Wang	8	43	33	16	143	1.43

2020-12-23	Wu	15	43	28	14	159	1.59
2020-12-23 11:19	Xu	10	33	35	22	131	1.31
2020-12-25 14:25	Zhang	8	48	30	14	150	1.5
2020-12-25 16:30	Yao	13	40	30	17	149	1.49
2020-12-28 11:43	Wang	15	45	30	10	165	1.65
2020-12-30	Tang	15	35	30	20	145	1.45
2020-12-30 12:59	Jiang	10	35	28	27	128	1.28
					median	149	1.49
					quartile (25%)	136.5	1.365
					quartile (75%)	159	1.59
